# Large-scale maps of altered cortical dynamics in early-stage psychosis are related to GABAergic and glutamatergic neurotransmission

**DOI:** 10.1126/sciadv.ads0400

**Published:** 2025-08-13

**Authors:** Ayelet Arazi, Alessandro Toso, Tineke Grent-‘t-Jong, Peter J. Uhlhaas, Tobias H. Donner

**Affiliations:** ^1^ Section Computational Cognitive Neuroscience, Department of Neurophysiology and Pathophysiology, University Medical Center Hamburg–Eppendorf, 20251 Hamburg, Germany.; ^2^ Department of Child and Adolescent Psychiatry, Charité Universitätsmedizin, 13353 Berlin, Germany.; ^3^ Institute of Neuroscience and Psychology, University of Glasgow, G12 8QB Glasgow, UK.; ^4^ Bernstein Center for Computational Neuroscience Berlin, Berlin, Germany.

## Abstract

Psychotic disorders affect GABAergic inhibition and glutamatergic excitation via NMDA receptors across the cerebral cortex. The mechanisms by which these distributed synaptic alterations produce the heterogenous symptoms of psychosis remain poorly understood. Using magnetoencephalographical source imaging, we mapped psychosis-related alterations of various features of intrinsic neural population dynamics across the human cortex. The cortex-wide patterns of these features were highly reproducible and related to the anatomical hierarchy of cortical areas. We found similar changes in these patterns for individuals with a first episode of psychosis and those at clinical high risk for psychosis. Maps of psychosis-induced changes in dynamics resembled the maps of GABA-A receptor densities and of pharmacological GABA-A or NMDA manipulation effects on cortical dynamics in healthy participants. The level of pattern similarity to GABA-A manipulation effects in individual patients correlated with positive symptoms, while the pattern similarity to NMDA effects correlated with negative symptoms. Our results open up a window on the distributed mechanisms of psychotic symptoms.

## Teaser

Cortex-wide changes in neural dynamics in early-stage psychosis relate to GABA-A or NMDA receptors and clinical symptoms.

## INTRODUCTION

Psychotic disorders, such as schizophrenia, present a major burden to society (*1*–*3*). Schizophrenia is characterized by positive and negative symptoms as well as by cognitive deficits (*4*). It is a neurodevelopmental disorder, with aberrations in brain circuitry emerging long before symptoms manifest, typically during adolescence (*3*). Criteria for the detection of individuals at clinical high risk for psychosis (CHR-P) have recently been developed (*5*). Approximately 25% of CHR-P individuals transition to psychosis within 3 years of follow-up (*6*).

The pathogenesis of psychotic disorders is complex and multifactorial, with aberrations manifesting at multiple levels of brain organization, and different symptoms possibly resulting from distinct mechanistic pathways (*4*). Several neurotransmitter systems are implicated in the underlying disease mechanism (*3*, *7*). Recent work has focused on GABAergic cortical inhibition (*8*–*11*) and glutamatergic excitation via *N*-methyl-d-aspartate (NMDA) receptors (*8*, *12*–*14*). Neural mass activity as measured during rest (*15*–*21*) or during sensory tasks (*22*, *23*) with electroencephalography (EEG) or magnetoencephalography (MEG) also exhibits marked alterations in psychotic disorders. The relationship between the alterations in specific neurotransmitter systems, local and large-scale cortical dynamics, and psychotic symptoms is currently not well understood. An important research goal, therefore, is to identify links between psychosis-related alterations at these different levels of analysis. Doing so may result in neurophysiological markers that are sensitive to disease mechanisms. This, in turn, may advance the early detection, diagnosis, and individualized treatment of psychotic disorders.

The microcircuits situated in different areas of the cerebral cortex share a common organization with intricate motifs of excitatory and inhibitory synaptic connections (*24*). The ratio between excitatory and inhibitory synaptic interactions, as well as their specific wiring motifs, jointly shapes core mechanisms of cognition, including persistent activity for working memory and the accumulation of evidence for deliberative decision-making (*24*–*32*). These microcircuit properties are disturbed in schizophrenia and related disorders, such as autism (*12*, *13*, *22*, *25*, *33*–*35*). Simulations show that changes in such microcircuit properties produce characteristic changes in the dynamics of spontaneous neural mass activity, as expressed in local field potentials (*36*–*42*). These neurophysiological mass signals, in turn, can be mapped with high spatiotemporal precision in the human brain using MEG source imaging (*15*–*21*, *43*, *44*)

While different regions of cortex share common circuit motifs, their properties vary across the cortex (*24*, *45*). One organizing principle underlying this variation is the hierarchical ordering of cortical areas (*24*, *46*) as defined by laminar anatomical connectivity patterns (*47*). In the human brain, an area’s position in the anatomical hierarchy can be inferred from the structural magnetic resonance imaging (MRI) T1w/T2w ratio, which tracks local myelin density (*48*) that is, in turn, inversely related to hierarchy (*46*). Like static circuit properties (e.g., the number of synaptic spines), the intrinsic timescales of cortical activity also seem to reflect the anatomical hierarchy (*24*, *39*, *49*–*53*). However, this link has not yet been quantified comprehensively across the entire human cortex (*39*, *50*). Last, mounting evidence points to a close link between the expression of neurotransmitter receptors and cortical dynamics (*53*), whereby anatomical hierarchy may again be a major underlying principle. For example, the pattern of the gene encoding the NMDA receptor subunit 2B increases with (T1w/T2w inferred) hierarchy (*46*), but the patterns of actual receptor expression may deviate from those of gene expression data (*54*). Positron-emission tomography (PET)–derived receptor expression maps, which resemble autoradiography data from non-human primates (*45*), correlate with the spatial distribution of features of cortical dynamics, such as frequency-specific neural oscillations (*52*, *53*).

The above insights into the large-scale organization of cortical circuits and cortical dynamics parallel the mounting evidence for the idea that the circuit dysfunctions underlying psychotic disorders are also expressed across many cortical areas (*3*, *22*). However, the large-scale organization of the distributed alterations in microscopic circuit properties has remained unknown. Likewise, it is unclear how such distributed alterations in circuit properties relate to changes in cortical dynamics and, ultimately, psychotic symptoms. Here, we developed an MEG source imaging approach to address these issues. We hypothesized that the large-scale patterns of altered cortical dynamics in psychosis result, at least in part, from the large-scale patterns of local alterations in GABA-A or NMDA receptors, which shape psychotic symptoms. Inspired by recent advances in computational neuroanatomy (*24*, *46*, *52*, *53*, *55*) and clinical neurophysiology (*56*, *57*), we aimed to (i) identify putative large-scale neurophysiological signatures of early-stage psychosis and (ii) quantitatively relate these signatures to large-scale patterns of GABA-A or NMDA receptor expression and function. To this end, we comprehensively mapped the large-scale patterns (*50*, *58*) of the changes of spontaneous cortical dynamics induced by psychosis or by pharmacological manipulations of GABA-A or NMDA receptors in healthy participants and tested for the reliability of, and relationship between, these large-scale patterns.

On the basis of results from invasive electrophysiology (*24*, *39*, *49*, *58*), we predicted that the local timescales of spontaneous cortical dynamics would be systematically related to the anatomical cortical hierarchy inferred from T1w/T2w ratios. We also expected the large-scale patterns of the changes in cortical dynamics induced by psychosis, or by pharmacological manipulations of GABA-A and NMDA receptors in healthy participants, to be widespread and heterogenous across cortex. Critically, because of the involvement of both GABA-A and NMDA receptors in the pathogenesis of psychosis, we predicted that different components of the psychosis-induced changes in cortical dynamics would resemble the pharmacologically induced changes in cortical dynamics. On the basis of the established interindividual variability of psychosis at the levels of molecules (*59*) and of circuit function (*3*), we also expected substantial individual differences between clinical participants in their expression of the changes in spatial patterns of cortical dynamics. Last, we asked whether and how these individual differences would relate to the individual expression of the positive symptoms (e.g., hallucinations or delusions) or the negative symptoms (e.g., lack of motivation or impairment of cognitive abilities such as attention or working memory) of psychosis. Previous accounts have related NMDA receptor hypofunction to cognitive impairment in schizophrenia (*13*) but little is currently known about the relationship between symptoms and GABA-A receptor function or the underlying alterations in cortical dynamics.

## RESULTS

We analyzed resting-state MEG data from two previously published studies (*15*, *60*–*64*). The clinical dataset was collected at the University of Glasgow in a group of CHR-P participants (*n* = 114), a group of patients with first-episode psychosis (FEP; *n* = 32), and a group of healthy controls (HCs; *n* = 45; see Materials and Methods for details on the samples). The pharmacological dataset was collected at the University Medical Center Hamburg-Eppendorf and measured the effects of double-blind oral administration of the GABA-A agonist lorazepam (LZP) and the NMDA receptor antagonist memantine (MEM), each compared to placebo within a group of 20 healthy participants (crossover design, with at least 1 week between successive sessions to allow for full elimination of the drugs; see Materials and Methods). All groups completed a block of eyes-open resting state, fixating a central cross on an otherwise blank screen.

### Mapping a multiparameter assay of neural population dynamics across cortex

**Fig. 1.**
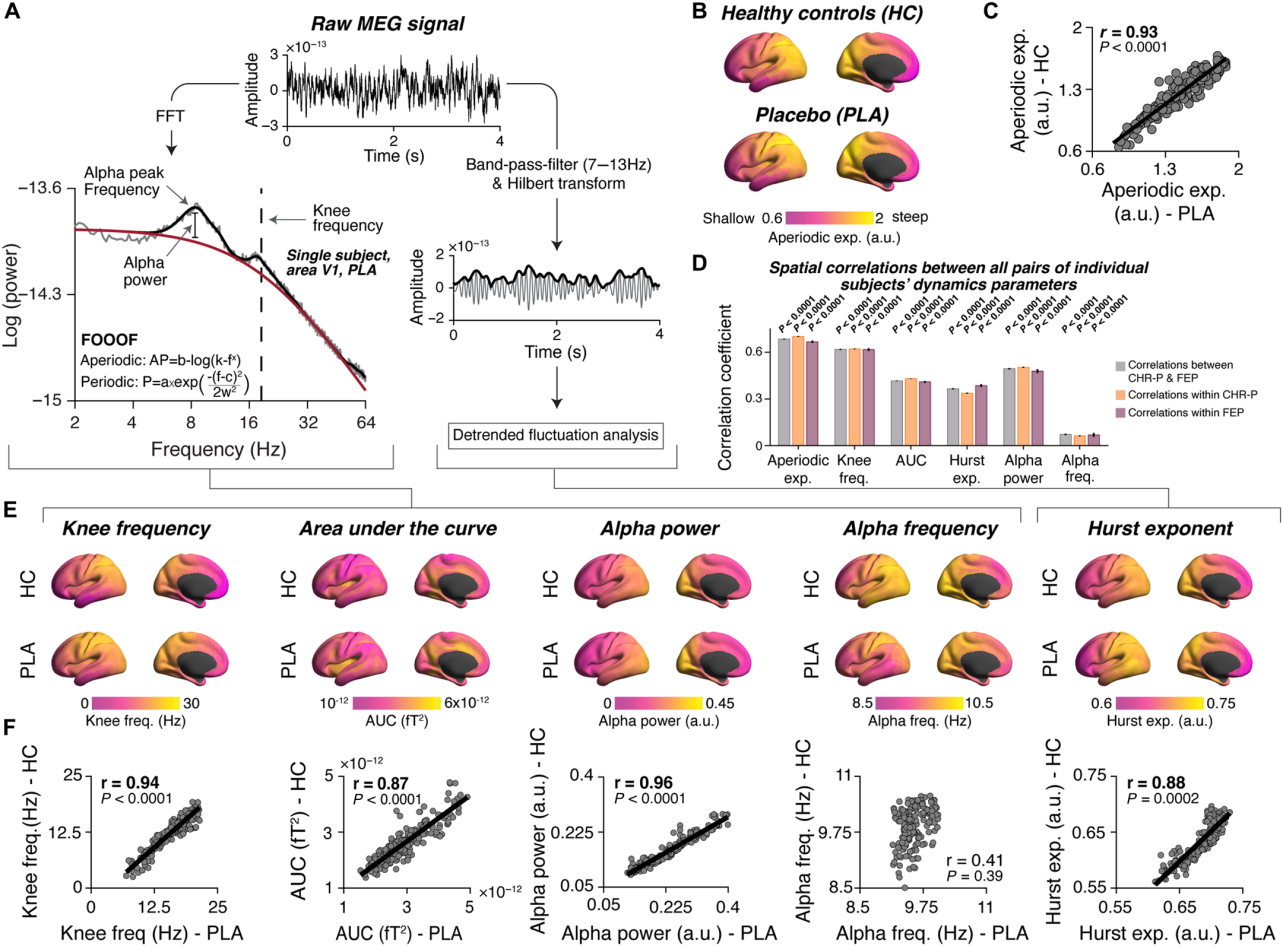
Large-scale spatial patterns of local cortical population dynamics. (**A**) Raw MEG signals (top; single participant, V1; placebo condition) were submitted to fast Fourier transform (FFT; left) or band-pass filtered and submitted to Hilbert transform (right). Left: Power spectral density (PSD; gray) of resting-state MEG signal. Black and red lines are the fitted periodic and aperiodic components, respectively (Materials and Methods). Right: Alpha (8 to 13 Hz) amplitude envelope of MEG signal were subjected to detrended fluctuation analysis to estimate the Hurst exponent (*66*). (**B**) Cortical spatial distribution of the aperiodic exponent across 180 areas. Here and in all following figures, homotopic regions from left and right hemispheres were averaged and results are presented on left hemisphere for illustration only. (**C**) Across-area correlations between the maps of aperiodic exponent in both samples shown in (B). (**D**) Across-area correlations between dynamics parameters maps of each pair of individual participants within each clinical group (orange; purple), and between clinical groups (gray). Error bars, SEM. (**E**) Cortical spatial distribution of five additional parameters of cortical dynamics. Top row, healthy control (HC). Bottom row, placebo condition (PLA). (**F**) Across-area correlations between maps of dynamics parameters of PLA condition and HC. Numbers in all scatter plots are Spearman correlation coefficients and *P* values (permutation tests; corrected for spatial autocorrelations). Data points, cortical areas; lines, linear fits. PLA, placebo condition; HC, healthy controls; CHR-P, clinical high risk for psychosis; FEP, first-episode psychosis.

We quantified spontaneous cortical network dynamics in the clinical and pharmacological datasets as a set of six parameters (Fig. 1 and Materials and Methods). The power spectra of local MEG signal fluctuations were decomposed into oscillatory (periodic) and nonoscillatory (aperiodic) components [Fig. 1A, left; (*65*)], from which we extracted a total of five parameters: the (i) exponent, (ii) “knee frequency,” and (iii) area under the curve (AUC; of aperiodic fit only) of the aperiodic component. We further extracted the (iv) power and (v) peak frequency of the periodic fit to the alpha (7 to 13 Hz) frequency range. In a separate analysis (Fig. 1A, right), we computed the so-called (vi) Hurst exponent of alpha-band amplitude envelopes to quantify long-range temporal correlations in the band power fluctuations (*66*).

We selected this set of parameters because each has been related to key circuit properties that may be altered in psychosis (*3*, *9*, *12*, *13*, *22*, *34*) and related mental disorders such as autism (*31*, *33*, *59*, *67*). The aperiodic exponent of the power spectrum and Hurst exponent of alpha-envelope fluctuations have both been linked to the ratio between local synaptic excitation and inhibition (*37*, *38*, *40*–*42*). The knee frequency is an inverse measure of the intrinsic timescale of cortical activity (*39*, *58*), which, in turn, depends on the strength of recurrent excitation (*24*). The AUC of the aperiodic fit is a proxy of population firing rates (*36*). Neural oscillations have been implicated as markers of excitatory-inhibitory interactions (*41*, *44*, *68*, *69*). While previous work on psychosis has focused on aberrations of gamma-band responses (*22*, *63*), we here focused on the alpha range, because alpha peaks are generally more prominent and widely distributed at rest (*44*), and were more consistently detected in our data across cortical areas and participants. However, the aperiodic parameters (i) to (iii) were based on the entire frequency range of the power spectra analyzed here, including the gamma range.

We comprehensively mapped this assay of six “dynamics parameters” across 180 well-defined areas covering the cortical surface (*55*, *70*). We averaged across corresponding areas from the left and right hemispheres and visualized the resulting parameter maps on the surface of the left hemisphere. This approach enabled us to characterize large-scale spatial patterns of changes in cortical dynamics in CHR-P and FEP groups versus control groups, or under pharmacological interventions versus placebo conditions in healthy individuals. The distribution of all dynamics parameters was heterogenous, exhibiting a smooth change across areas (Fig. 1). The maps of all parameters except for the AUC had the highest values in occipital areas (yellow in Fig. 1, B and E) and the lowest values in frontal areas (pink).

Several analyses indicated that these large-scale spatial patterns were reliable features of cortical dynamics. Maps of the aperiodic exponent strongly correlated between independent samples of participants [HC versus participants of pharmacological study (placebo condition); Fig. 1C: *r*(180) = 0.93 and *P* < 10^−4^]. The maps of the other parameters (Fig. 1E), likewise, exhibited robust correlations between both samples (Fig. 1F). Furthermore, the maps of the different parameters were robustly correlated among one another (fig. S1, A and B). The spatial patterns of dynamics parameters were also correlated between individuals, within and between clinical groups (Fig. 1D). Further, patterns of parameters tended to peak (both positive and negative peaks) at approximately the same cortical areas across individual participants, within the HC or placebo condition samples (fig. S1C). In sum, the spatial distributions of dynamics parameters were consistent across individual participants within samples and across distinct clinical samples.

### Large-scale patterns of cortical dynamics reflect cortical hierarchy

**Fig. 2.**
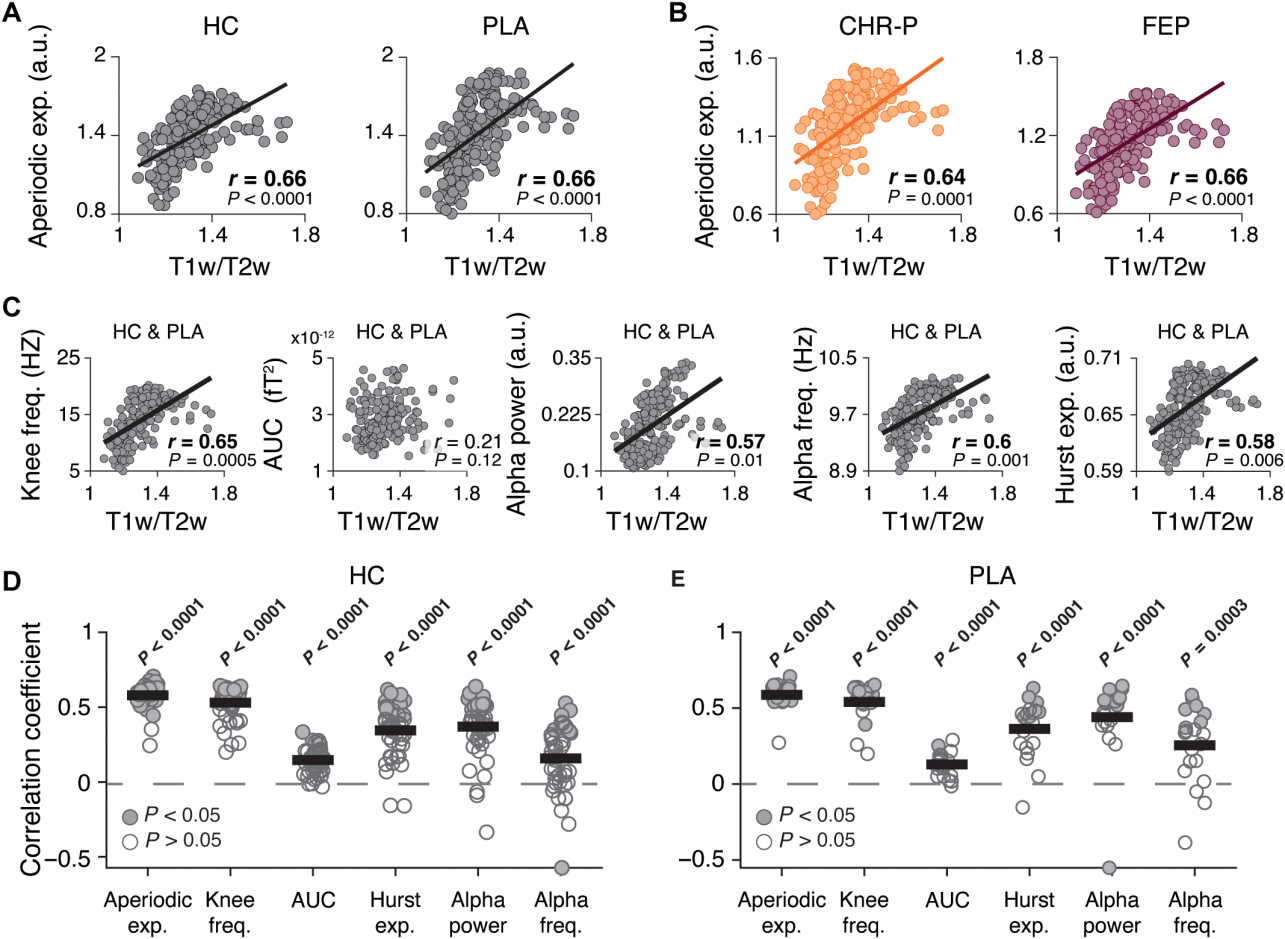
Spatial patterns of cortical population dynamics reflect cortical hierarchy. (**A**) Across-area correlations between maps of aperiodic exponents and T1w/T2w MRI ratio (MRI maker of anatomical hierarchy, see main text). Data points, cortical areas; line, linear fit; numbers are Spearman correlation coefficients and *P* values (spatial autocorrelation-preserving permutation test). (**B**) Same as (A), for the two clinical groups. (**C**) Same as (A), for other dynamics parameters. (**D** and **E**) Across-area correlations between maps of dynamics parameters and T1w/T2w ratio for individual participants. Data points (circles), participants; filled circles, statistically significant correlation coefficients (permutation tests, corrected for spatial autocorrelations). PLA, placebo condition; HC, healthy controls; CHR-P, clinical high risk for psychosis; FEP, first-episode psychosis.

As expected, the large-scale spatial patterns of cortical dynamics reflected the spatial maps of the structural MRI T1w/T2w ratio (proxy of cortical hierarchy; Fig. 2 and fig. S2). We found that the spatial patterns of the exponent of the aperiodic component were both positively correlated with the T1w/T2w ratio in the control groups or conditions of both datasets [Fig. 2A: *r*(180) = 0.66 and *P* < 10^−4^; spatial autocorrelation-preserving permutation tests; Materials and Methods]. In other words, the exponent of the aperiodic component decreased across the cortical hierarchy, with large exponents (i.e., steep decay of power with frequency) for early sensory cortices and small exponents (i.e., shallow decay) for prefrontal cortex. The aperiodic exponent has been proposed as a noninvasive proxy of the local excitation-inhibition ratio (*38*), which, in turn, increases as a function of cortical hierarchy (*24*). Likewise, the maps of the knee frequency [inverse of neural timescales (*39*, *58*)] showed a decrease of knee frequencies from sensory to association cortex (Fig. 2C), in line with slower neural timescales in higher-tier areas of the primate cortex (*24*, *49*, *50*).

The maps of most other parameters (Fig. 1E) were, likewise, correlated with the T1w/T2w ratio maps in both datasets (Fig. 2C and fig. S2, A and B), in both clinical groups and pharmacological conditions (Fig. 2B and fig. S2, A and B). These correlations were also significant in most individual participants of both samples (Fig. 2, D and E).

The above results replicate the finding from previous invasive recordings (*39*, *49*), that the intrinsic timescale of neural activity reflects the cortical hierarchy. Our results go beyond previous findings in showing that the cortex-wide spatial patterns of cortical dynamics are highly reproducible across independent samples (and even different individuals) obtained in separate MEG laboratories, and their relation to cortical hierarchy generalizes to several parameters of cortical dynamics and is preserved under both rapid (within-session) pharmacological perturbation of synaptic transmission and a protracted clinical aberration (early stage psychosis). Thus, the relationship between neural dynamics and cortical hierarchy is a robust feature of the cerebral cortex.

### Reproducible cortex-wide patterns of psychosis-related changes in neural dynamics

We went on to assess the spatial maps of the differences in dynamics parameters between CHR-P or FEP groups versus controls and between the drug versus placebo conditions. We computed the difference maps between the average maps for each of the two clinical groups (CHR-P or FEP) versus the HC group. In the following, we refer to the parameter difference maps between the clinical groups (CHR-P or FEP or both pooled) versus the HC group as “psychosis signatures.” We defined the psychosis signatures in terms of the difference maps rather than the maps of raw parameters in the clinical groups, to isolate the patterns specific to psychosis (the raw maps were similar in groups and largely explained by cortical hierarchy in both; figs. S1A and S2). GABA-A and NMDA receptors (*53*, *71*, *72*) as well as the circuit dysfunctions in schizophrenia (*3*, *22*) are expressed across many cortical areas, but with heterogeneous distribution, possibly over and above the large-scale gradient determined by anatomical hierarchy. We, therefore, reasoned that the psychosis signatures of the clinical populations might exhibit characteristic large-scale spatial patterns.

**Fig. 3.**
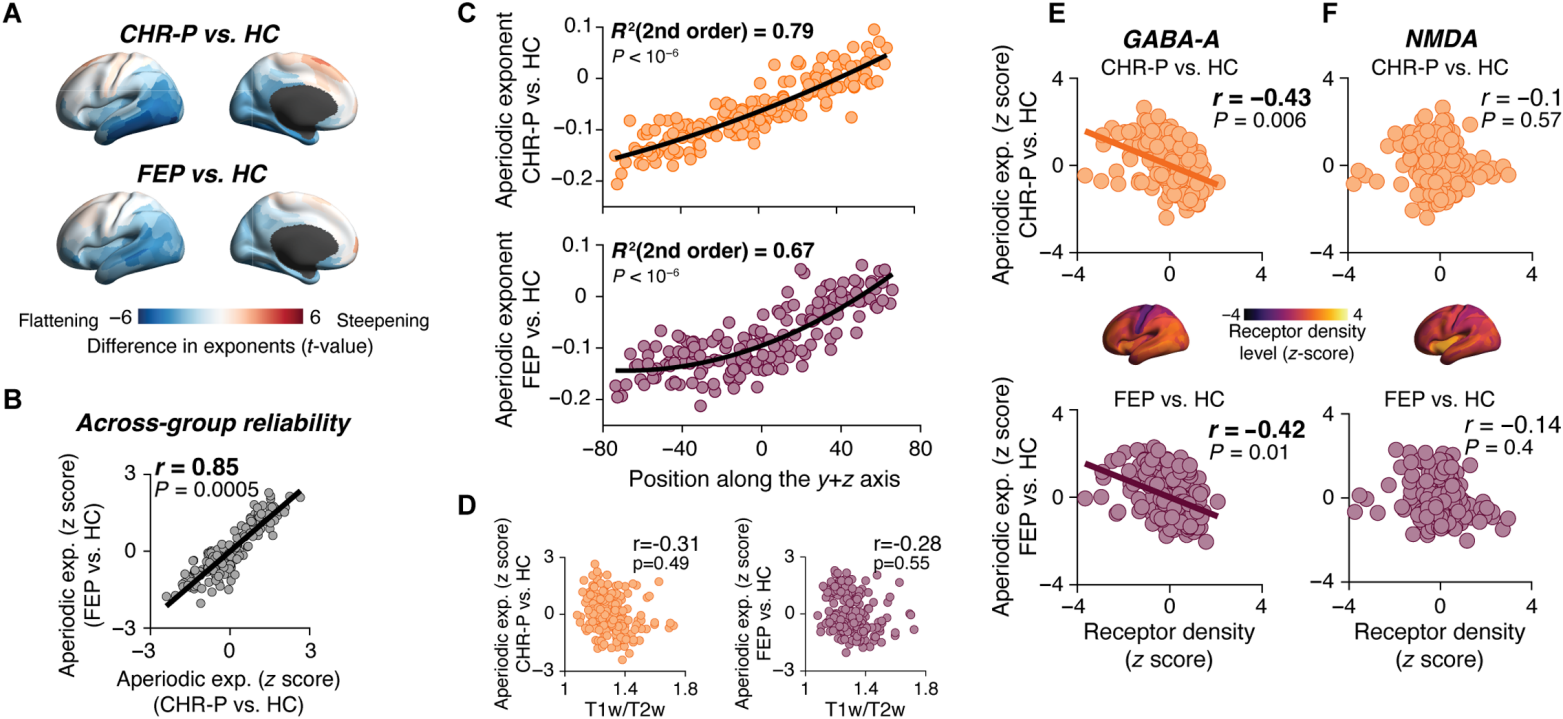
Reproducible spatial pattern of psychosis signature. (**A**) Map of psychosis signature for aperiodic exponent, computed as group-level *t* score maps (shown here without threshold) of the difference between the exponents for the respective clinical group versus HC group. (**B**) Correlation between psychosis signatures (aperiodic exponent) in both clinical groups. (**C**) Relation of psychosis signature (aperiodic exponent) with position on the *y* + *z* axis in the anatomical MNI standard space. Data points, cortical areas; black line, second-order polynomial fit; *R*^2^ and *P* values of the best-fitting model (second-order polynomial) from *F* test (Materials and Methods). (**D**) Correlation between psychosis signatures (aperiodic exponent) and T1w/T2w ratio maps. (**E** and **F**) Correlation of psychosis signature (aperiodic exponent) and maps of GABA-A (D) or NMDA (E) receptor densities (shown in insets) for both clinical groups (top/orange; bottom/purple). Receptor density maps were from reference (*53*). Data points, cortical areas; lines, linear fit; numbers are Spearman correlation coefficients and *P* values (spatial autocorrelation-preserving permutation tests). PLA, placebo condition; HC, healthy controls; CHR-P, clinical high risk for psychosis; FEP, first-episode psychosis.

We show the difference maps for the aperiodic exponent in Fig. 3A and for all other parameters in fig. S3 (A to E). Exponents tended to be smaller for the clinical groups compared to the HC group in occipital, temporal, and parietal areas including the visual and auditory cortices (blue in Fig. 3A), indicating a flattening of the decay of power with frequency. This is in line with an increase in the local E/I ratio as has been postulated for schizophrenia (*13*, *22*, *34*). However, exponents in frontal cortical areas, especially around the midline, tended to be larger in the clinical groups indicating a steepening of the power decay (red in Fig. 3A), in line with some EEG results (*16*, *73*). In other words, the psychosis signature exhibited heterogeneity across cortex for the aperiodic exponent (Fig. 3A). The same was true for other parameters (fig. S3, A to E).

For the aperiodic exponent, the psychosis signature varied smoothly from the posterior-ventral to the anterior-dorsal cortex (Fig. 3A), a pattern not evident for other parameters (fig. S3, A to E). Areas’ values in the psychosis signature (i.e., differences in exponent) were most strongly related to their position on the posterior-ventral to anterior-dorsal (i.e., *y* + *z* diagonal of the MNI standard space; Fig. 3C and Materials and Methods). Relationships to position on other axes were weaker (fig. S4). Notably, the local difference scores evident in the (nonthresholded) maps of parameter changes were subtle (see the “Weak local effects of psychosis or pharmacology” section). Here, we focused on the spatial patterns of parameter changes, asking whether they were reliable and informative about disease mechanism and clinical status.

We asked whether these spatial patterns were consistent across both clinical groups. The spatial patterns of parameter changes were strongly correlated between the CHR-P and FEP samples [*r*(180) = 0.85 and *P* = 0.0005; Fig. 3B]. Similar pattern consistency between clinical groups was observed for the changes in the other parameters (fig. S3F). In other words, the spatial patterns of parameter changes shown in Fig. 3A are stable pathophysiological markers of psychosis that are already detectable in a prediagnostic (CHR-P) stage, supporting the use of the term “psychosis signature” to refer to these spatial patterns.

The psychosis signature was, for most parameters, not significantly related to anatomical hierarchy (Fig. 3D and fig. S5C), but it instead resembled the cortical distributions of neurotransmitter receptor densities (Fig. 3, E and F; see receptor maps in insets): Areas exhibiting a “flattening” of power spectra in psychosis (blue in Fig. 3A, smaller exponents) tended to have higher-than average GABA-A receptor densities, and conversely for areas with a “steepening” (red in Fig. 3A) of power spectra (Fig. 3E). We found no significant correlation for NMDA receptor density map for either clinical group (Fig. 3F), although GABA-A and NMDA receptor density maps were weakly correlated with one another [*r*(180) = 0.24 and *P* = 0.02].

Except for alpha power (GABA-A receptor densities versus FEP signatures), and alpha peak frequency (NMDA receptor densities versus CHR-P and FEP signatures), correlations with receptor density maps were not evident for the other dynamics parameters (fig. S5, A and B). The smaller correlation with NMDA compared to the GABA-A receptor density maps was not due to a difference in across-cortex variation, as both maps had similar statistical properties (spatial variance, GABA-A: 0.95 and NMDA: 0.93; spatial autocorrelation Moran’s I, GABA-A: 0.074 and NMDA: 0.06; see Materials and Methods).

Together, these results are in line with the idea that the distributions of the densities of GABA-A receptor contribute to the spatial patterning of the neurophysiological psychosis signature. In other words, the spatial patterns of the change in the aperiodic exponent in CHR-P and FEP-groups compared to the HC group reflect the spatial distribution of neurotransmitter receptors controlling excitatory-inhibitory interactions (in particular GABA-A receptors) in the healthy brain.

### Classification of clinical groups based on large-scale dynamics

An important issue from the perspective of biomarker development is the diagnostic value of the spatial patterns of neurophysiological markers that we analyzed here. We trained multivariate pattern classifiers to predict clinical status based on the individual spatial patterns of dynamics parameters (Materials and Methods). We found above-chance classification of the clinical status of individual participants whose data were not used for training, for both comparisons, FEP versus HC and CHR-P versus HC (Table 1). This was true for classifiers that used the spatial patterns containing all six parameters as features, as well was for classifiers that only used the maps of changes in aperiodic exponent (as a proxy of excitation-inhibition ratio, see above), or of the aperiodic exponent and knee frequency (proxy of neural timescale; Table 1). As expected from the large similarity of the psychosis signatures measured in the two clinical groups (Fig. 3B), classification of CHR-P versus FEP was not possible (Table 1).

**Table 1.** Accuracy of classifying clinical groups based on patterns of dynamics parameters. Classification accuracies are cross-validated and *P* values from permutation testing (see Materials and Methods for details). *P* values of classification accuracies are from two-sided permutation tests across participants; CI, confidence interval, based on 200 random samples (see Materials and Methods).

Parameter combination	Classification problem	Fraction of correct classifications (*P* value)	Area under ROC (66% CI)	F1 score
Aperiodic exponent only	CHR-P vs. HC (*n* = 45)	0.66 (**0.007**)	0.67 (0.61–0.73)	0.66
	FEP vs. HC (*n* = 32)	0.63 (**0.044**)	0.56 (0.47–0.68)	0.63
	CHR-P vs. FEP (*n* = 32)	0.62 (0.065)	0.53 (0.37–0.69)	0.63
Aperiodic exponent & knee frequency	CHR-P vs HC (*n* = 45)	0.67 (**0.007**)	0.66 (0.60–0.73)	0.67
	FEP vs. HC (*n* = 32)	0.62 (0.057)	0.58 (0.51–0.68)	0.63
	CHR-P vs. FEP (*n* = 32)	0.56 (0.182)	0.40 (0.20–0.56)	0.55
All 6 parameters	CHR-P vs HC (*n* = 45)	0.65 (**0.011**)	0.65 (0.57–0.72)	0.65
	FEP vs. HC (*n* = 32)	0.61 (0.089)	0.53 (0.44–0.63)	0.63
	CHR-P vs. FEP (*n* = 32)	0.54 (0.298)	0.34 (0.14–0.53)	0.56

While the classification of CHR-P was more accurate, and highly statistically significant, for all combinations of features, classification of FEP was overall less accurate for all combinations and only statistically significant for the classifier based on exponent maps (marginally significant for the ones using combinations of parameter maps; Table 1). This difference could reflect the smaller size of the FEP sample (*n* = 32 for FEP versus HC classifier, versus *n* = 45 for CHR-P versus HC classifier; see Materials and Methods), the fact that a subset of FEP (but no CHR-P) participants were treated with antipsychotics (*62*, *63*), or a combination of both factors. In line with the first account, repeating the CHR-P versus HC classifications using randomly drawn subsamples of *n* = 32 reduced the classification accuracy [and receiver operating curve (ROC)–AUC values, see below] to values closer to the FEP versus HC classification.

From the continuous outputs of the classifiers (see Materials and Methods), we also calculated the area under the ROC (*74*) that quantifies the overlap of the distributions of these classifier outputs. The resulting ROC values can be interpreted as the probabilities that a randomly chosen individual is correctly identified as belonging to the CHR-P or FEP groups versus the HC group. ROC values ranged between 0.53 and 0.67 for the different comparisons of the CHR-P versus HC or FEP versus HC, performed using different feature combinations (Table 1; again, lower ROC values for CHR-P versus FEP classification). The F1 scores, which were based on the classifier confusion matrices (fig. S6 and Materials and Methods) and combined both precision and sensitivity, ranged from 0.63 to 0.67 for all comparisons except for CHR-P versus FEP (Table 1). Overall, the classification results highlight the potential utility of the here-identified psychosis signatures as part of an assay for the early detection of psychosis.

### Reproducible patterns of pharmacologically induced changes in neural dynamics

**Fig. 4.**
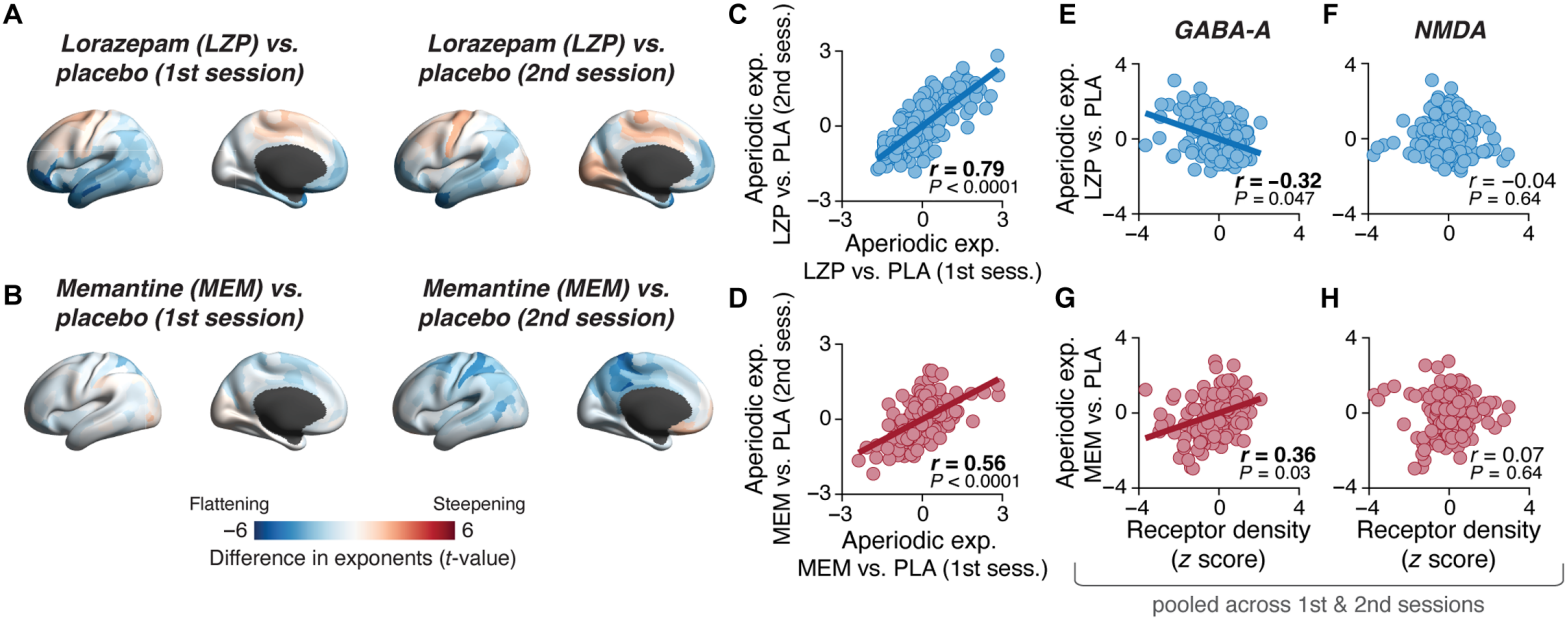
Reproducible spatial patterns of drug effects on exponent of aperiodic activity. (**A** and **B**) Maps of drug effects on the aperiodic exponent (group-level, nonthresholded *t* maps) for the first (left) or second (right) repetition of each drug condition across different sessions. (A), lorazepam (LZP); (B), memantine (MEM). (**C** and **D**) Spatial similarity of drug effects in the first versus second administration of each drug condition, a measure of test-retest reliability. (C), LZP; (D), MEM. (**E** and **F**) Spatial similarity of map of LZP effects on aperiodic exponent and maps of GABA-A (E) or NMDA (F) receptor densities. (**G** and **H**) As (E) and (F), but for map of MEM effects on aperiodic exponent. Throughout, *r* values are Spearman’s correlation coefficients, *P* values were assessed using spatial autocorrelation-preserving permutation tests, and lines are linear fits for significant correlations.

We next assessed whether and how the neurophysiological psychosis signatures identified above related to the changes in cortex-wide dynamics induced by pharmacological manipulations of GABA-A and NMDA receptors in healthy participants. Administration of LZP (GABA-A receptor stimulation) and MEM (NMDA receptor blockade) produced widespread changes in the aperiodic exponent compared to placebo. In the following, we refer to the corresponding difference maps as “drug effect maps.” Again, we show the difference maps for the aperiodic exponent in Fig. 4, A and B and for all other parameters in fig. S3 (A to E). Like the psychosis signatures, the drug effects varied across cortical areas, with smaller exponents (“flattening of PSDs,” blue in Fig. 4, A and B) in some areas and larger exponents in other areas (“steepening of PSDs,” red in Fig. 4, A and B). Similar heterogeneity was observed in the drug effect maps for other dynamics parameters (fig. S3, A to E).

These drug effect maps again exhibited stable and reproducible patterns, which reflected the distribution of synaptic properties (Fig. 4, C to H). The pharmacological study entailed two MEG sessions for each pharmacological condition (separated by 1 to 5 weeks; Materials and Methods). The spatial patterns of both the effects of both drugs were largely consistent across these two condition repeats for the aperiodic exponent (Fig. 4, C and D) as well as on most of the other parameters (fig. S3, G and H). As expected, the map of the LZP effects on exponent (but not the other parameters) exhibited significant spatial similarity to the map of the density of the GABA-A receptors that are targeted by this manipulation (Fig. 4E), but not to the map of NMDA receptor densities (Fig. 4F). Unexpectedly, the map of MEM effects on exponent also correlated (with opposite sign) with GABA-A receptor densities (Fig. 4G; no correlation with map of NMDA receptor densities).

These results establish the reproducibility and importance of the large-scale spatial patterns of changes in cortical dynamics induced by the two drugs. They also reveal a notable similarity in the spatial patterns of the average LZP effect and average psychosis signatures (e.g., compare Figs. 4A and 3A), an impression that we quantify systematically below (see the “Relating changes of neural dynamics induced by psychosis or pharmacological manipulations” section).

### Weak local effects of psychosis or pharmacology

**Fig. 5.**
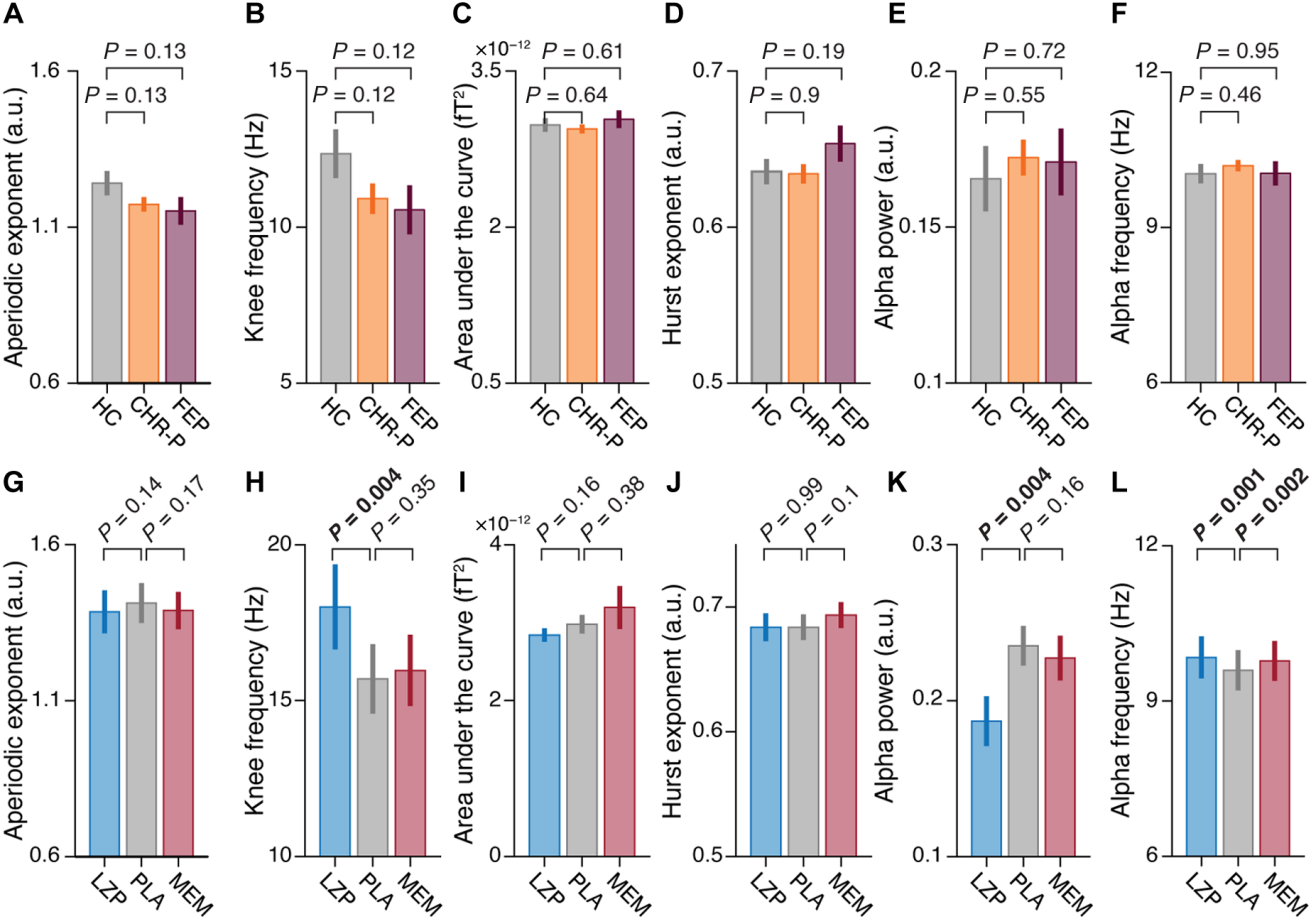
Weak local effects of psychosis or drugs on cortical dynamics. (**A** to **F**) Means and SEs across all 180 parcels for healthy control (HC; gray), clinical high risk (CHR-P; orange), and first-episode psychosis (FEP; purple) groups of the aperiodic exponent (A), knee frequency (B), area under the curve (C), Hurst exponent (D), alpha power (E), and alpha peak frequency (F). (**G** to **L**) Same as (A) to (F) for the pharmacological conditions; LZP, lorazepam (blue); PLA, placebo (gray); MEM, memantine (red). All *P* values were assessed with two-sided *t* tests.

Our analyses of the cortex-wide spatial patterns of changes in cortical dynamics induced by early stage psychosis or pharmacological manipulations were motivated from the emerging understanding of the characteristic patterns of receptor expression in the healthy brain (*46*) as well as of circuit aberrations in schizophrenia (*3*). The observations that the psychosis signatures and drug effects in cortical dynamics exhibited stable and diagnostic spatial patterns across cortex further corroborated our focus on the spatial patterns. Control analyses complemented the pattern analyses by conventional mass-univariate statistical tests of the local changes in the parameters shown in Figs. 3A and 4 (A and B) and fig. S3 (A to E). At the granularity of the original 180 parcels used throughout these maps, apart for a widespread effect of LZP on alpha power, no local differences were statistically significant after rigorous false discovery rate correction for multiple comparisons (fig. S7, A to F). To boost statistical sensitivity, we aggregated each dynamics parameter across areas before comparing between clinical groups or pharmacological conditions. We aggregated at two levels of granularity: (i) across all parcels belonging to each of 22 groups of areas (*55*) (fig. S7, G to L) and (ii) across the complete set of 180 areas, yielding a single number per parameter (psychosis signatures: Fig. 5, A to F; drug effects, Fig. 5, G to L). Even at these coarser levels, few comparisons were statistically significant (Fig. 5 and fig. S7), especially the clinical datasets that yielded only two significant effects [in the ventral stream visual cortex and (FEP and CHR-P) and medial temporal cortex (CHR-P) (*55*) for the area under the aperiodic curve; fig. S7I]. Bayes factors consistently provided strong support for the null hypothesis [i.e., ${\text{BF}}_{10}$ < 1/3 (*75*)] for most comparisons in the clinical dataset [HC versus CHR-P or FEP; AUC: ${\text{BF}}_{10}$ < 0.27; Hurst exponent: ${\text{BF}}_{10}$ = 0.19 (CHR-P only); alpha power: ${\text{BF}}_{10}$ < 0.25; and alpha frequency: ${\text{BF}}_{10}$ < 0.24].

In sum, the local effects of psychosis (CHR-P or FEP) or of pharmacological interventions (LZP or MEM) were not sufficiently strong to be reliably detectable using mass-univariate statistical testing when correcting for multiple comparisons. At the same time, the above pattern analyses demonstrate that these weak local changes were not just noise: The spatial patterns of these local changes (i) were reliable across groups (Fig. 3B) or repeated sessions (Fig. 4, C and D), (ii) correlated with receptor densities (Figs. 3E and 4, E and G), and (iii) enabled clinical classification, especially for the CHR-P group (Table 1). Together, these results highlight the power of spatial pattern analyses for identifying alterations in spontaneous brain dynamics due to psychosis and their relation to neurotransmitter systems (see Discussion).

### Relating changes of neural dynamics induced by psychosis or pharmacological manipulations

**Fig. 6.**
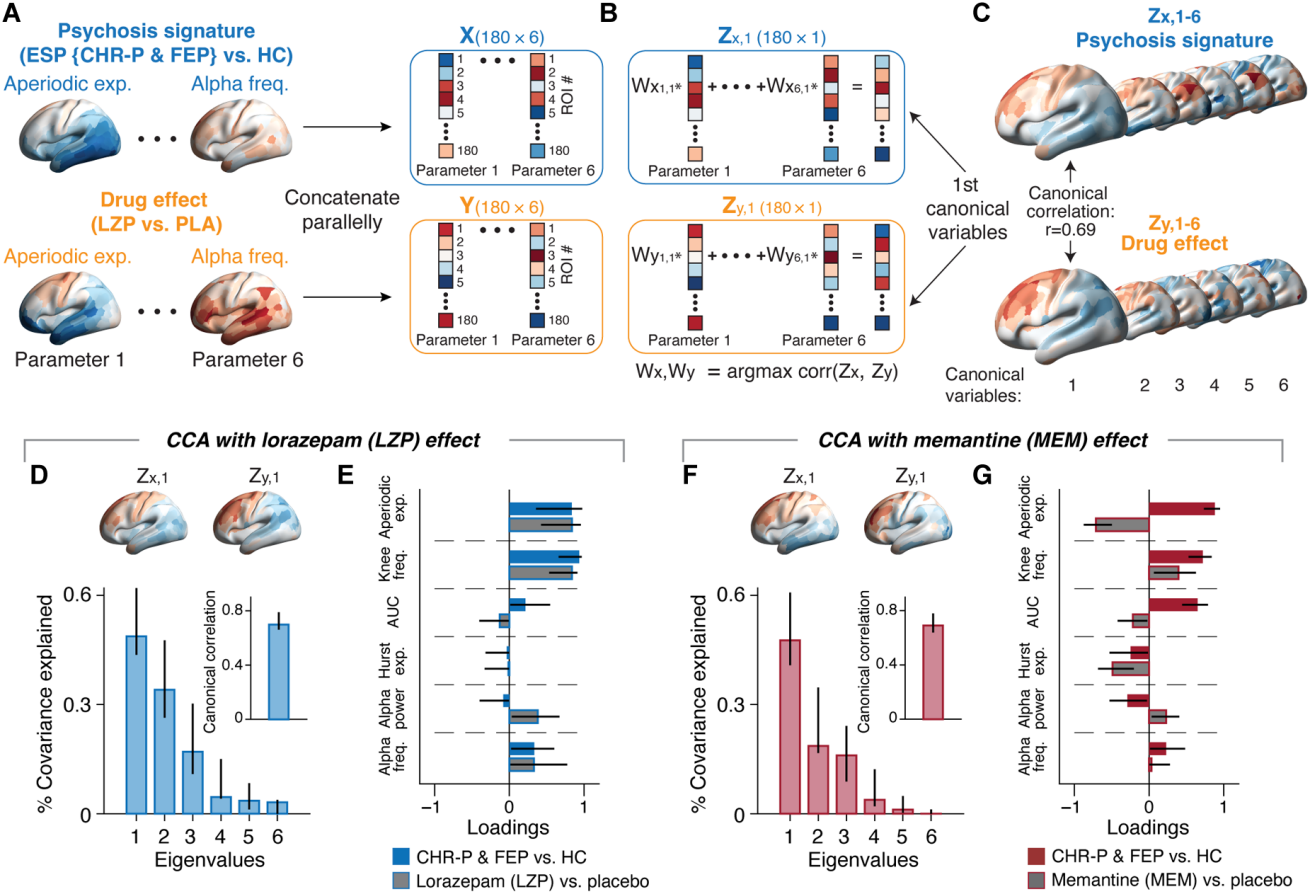
Exponent and knee frequency dominate similarity between psychosis signatures and drug effects. (A to C) Schematic illustration of canonical correlation analysis (CCA). (**A**) Maps of early-stage psychosis (ESP) signatures or drug effects (left, for exponent and alpha frequency) were concatenated parallelly across the six-parameter set to form two matrices Xand Y , respectively, each with 180 rows (areas) and 6 columns (parameters; right). (**B**) CCA determined 6 × 6 matrices of linear weights ( $W_{x},W_{y}$ ). Multiplication of these weight matrices with *X* and *Y*, respectively ( $Z_{x}=XW_{x}\;\text{and}\;Z_{y}=YW_{y}$ ), yielded canonical variables (six column vectors of $Z_{x}\;$and $Z_{y}$ ) that maximized the spatial correlation. (**C**) Maps of canonical variables $Z_{x,i}\;$and $Z_{y,i}$ , *i* = 1…6. (**D** and **E**) Results of LZP-CCA. (**F** and **G**) Results of MEM-CCA. (D) and (F) show the spectra of eigenvalues quantifying the fraction of covariance between *X* and *Y* explained by each pair of canonical variables. Insets: canonical correlation coefficients of the first canonical variables. Top: maps of the first canonical variable. (E) and (G) show the CCA loadings reflecting the relative contribution of each parameter to the canonical correlation. Error bars are 95% bootstrap confidence interval.

We went on to quantify the similarity between the spatial patterns of changes induced by psychosis (psychosis signature) and by the pharmacological interventions in healthy participants, using two complementary approaches. The first approach was canonical correlation analysis (CCA), a multivariate statistical technique that determines the linear combinations of two sets of random variables (i.e., matrices) that maximizes the correlation between the two. Here, the random variables were the spatial maps of psychosis signatures or drug effects in the six cortical dynamics parameters defined in Fig. 1. We horizontally concatenated these spatial maps, yielding two matrices with 180 rows (areas) and six columns (parameters; Fig. 6A). The variables resulting from the linear transformations are called “canonical variables” (Fig. 6, B and C): sets of spatial maps (for psychosis signatures and drug effects), which were ordered by the fraction of covariance explained by each pair of variables (Fig. 6C). We performed two separate CCAs for the LZP and the MEM effects, respectively. In each case, we used a single psychosis signature pooled across the two clinical groups (CHR-P and FEP). We refer to these pooled data as “early-stage psychosis (ESP)” for simplicity. Pooling the data across clinical groups was justified by the strong and significant similarity of the patterns for both groups shown in Fig. 3B (correlation of 0.85 for exponent) and fig. S3F (correlations between 0.57 and 0.75 for the other parameters).

The first set of canonical variables accounted for a substantial fraction of the covariance shared between the psychosis signature and drug effect: 48% for LZP (Fig. 6D) and 47% for MEM (Fig. 6F), each more than 10% larger than the covariance explained by the next set of canonical variables. We, therefore, focused on the first canonical variables. These yielded a strong canonical correlation of *r*(180) = 0.69 for both drug effects (Fig. 6, D and F, inset). Only the magnitude, not the sign, of the canonical correlations were meaningful because canonical correlates are positive by construction.

The loadings of the canonical variables quantified the relative contribution of the six parameters of cortical dynamics to the canonical correlation. Under LZP, the loadings were the largest for the aperiodic exponent and knee frequency, each with equal sign for psychosis signature and LZP effect (Fig. 6E), indicating that the spatial patterns of the changes of both parameters due to LZP and psychosis were positively correlated. This supports the visual impression that the maps of changes in exponent in both clinical groups (Fig. 3A) and under LZP (Fig. 4A) resembled one another. By contrast, under MEM, the aperiodic exponent had large loadings of opposite sign between drug effect and psychosis signature (Fig. 6G), indicating an anticorrelation between their respective spatial patterns, again supporting the visual impression from comparing Figs. 3A and 4B.

Akin to the maps of the psychosis signatures (Fig. 3) and drug effects (Fig. 4), the maps of canonical variables were highly reproducible across independent data sets (fig. S8). Consider the similarity of the two canonical variables between repeated sessions for a version of the LZP-CCA (fig. S8A). Correlations between the maps within each row are the corresponding canonical correlations, while the correlations between the canonical variable maps within each column quantify their consistency across the two clinical samples (drug effects pooled across repeats of each drug condition). We ran each CCA separately for the psychosis signature patterns in the CHR-P and FEP groups and separately for the drug effect maps in the two sessions of each drug condition. For each of the LZP-CCAs and MEM-CCAs, this yielded a total of four pairs of first canonical variables (Materials and Methods): two pairs of maps for the clinical groups times two pairs of maps for the session repeats. These maps exhibited substantial spatial similarities [*r*(180) ≥ 0.28], most (21 of 24) of which were statistically significant after spatial autocorrelation-preserving permutation tests (fig. S8, E and F).

The loading patterns were, likewise, reasonably consistent across independent data sets, albeit less so than the maps of canonical variables. Figure S8 (C and D) shows their similarity across the two clinical samples (drug effects pooled across repeats of each drug condition), with high and significant correlations for the LZP-CCA (not the MEM-CCA). Many correlations of the loading patterns across the different repeats of CCAs were statistically significant (13 of 24; 2 further marginally significant at *P* = 0.06), but more consistently for LZP than for MEM (fig. S8, G and H).

Together, the CCA approach established substantial large-scale pattern similarity between the psychosis signatures of the CHR-P and FEP groups on the one hand and drug effects on the other hand. These similarities were consistent across two clinical groups and two separate experimental sessions in healthy participants several weeks apart. The approach also uncovered the relative contribution of the six dynamics parameters to this pattern similarity, highlighting the contributions of the exponent and knee frequencies of the aperiodic activity components.

### Individual differences in similarity levels account for clinical symptoms

CCA is a principled approach to relating multivariate sets of spatial patterns. Here, CCA shed first light on the spatial similarity relationships between psychosis signatures and drug effects as well as the dynamics parameters driving those similarities. However, our CCA approach had two limitations: It required (i) a focus on the relationships captured by a subset (in our case the first) of the canonical variables and (ii) averaging the psychosis signatures across the groups of participants. We reasoned that the latter limitation may be important with respect to psychosis, a condition known to exhibit considerable interindividual variability, at the genetic and molecular levels (*59*) as well as at the level of circuit function (*3*). In other words, different patients diagnosed with, or at risk of developing psychosis might exhibit distinct spatial patterns of neurotransmitter alterations. Thus, we reasoned that their neurophysiological psychosis signatures may also vary.

**Fig. 7.**
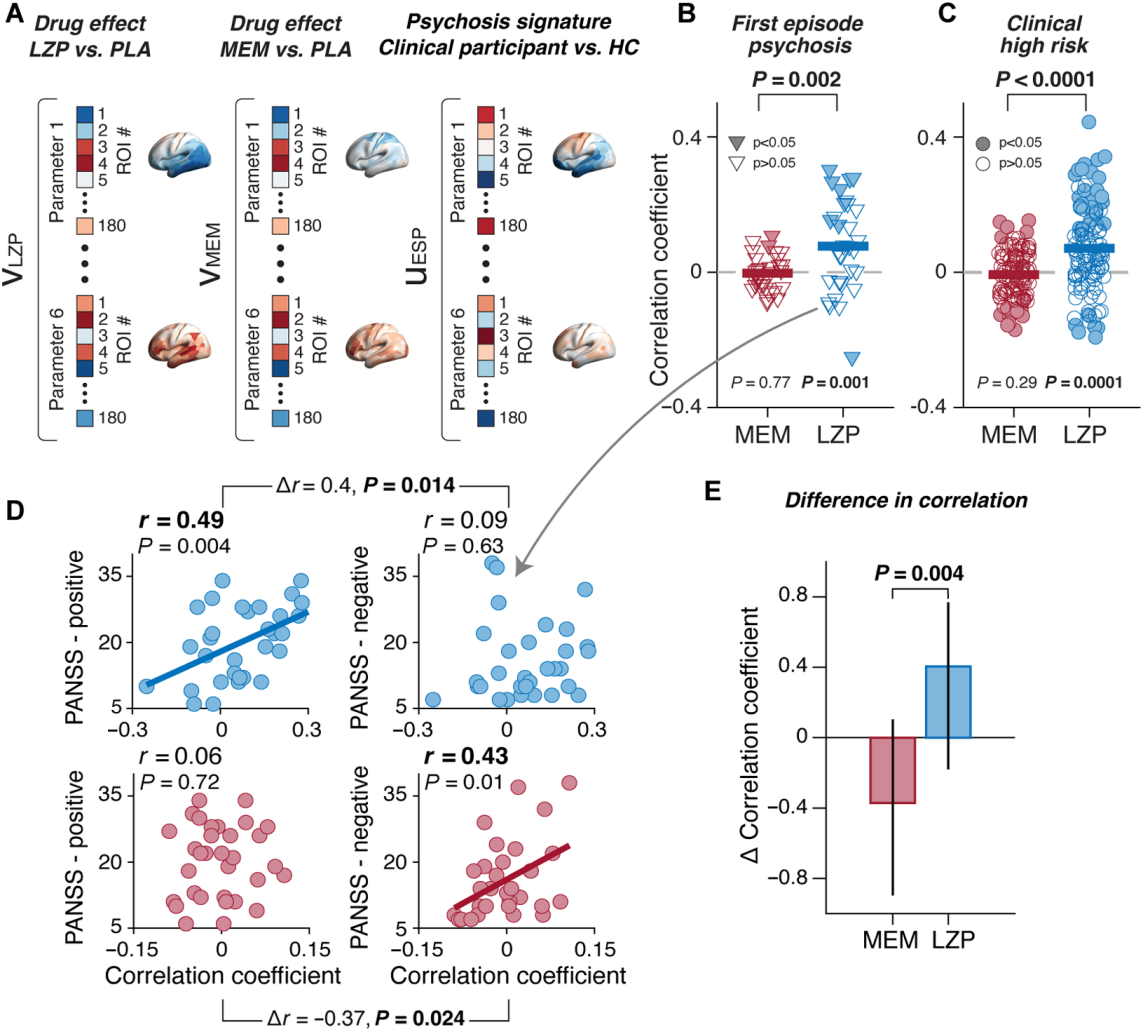
Individual differences of pattern similarities to drug effects relate to clinical symptoms. (**A**) Schematic illustration of individual difference analysis approach. Maps of the drug effect or psychosis signature (column vectors with 180 elements) were concatenated vertically across the six parameters to form column vectors with 1080 elements (6 parameters × 180 areas). (**B**) Spatial similarity between group average drug effects and individual psychosis signatures (FEP versus HC average). Data points, correlation of individual-patient psychosis signature and a given drug effect (MEM, red; LZP, blue). Filled symbols, individual patients with statistically significant pattern correlation. (**C**) Same as (B) for individual psychosis signature of CHR-P (CHR-P versus HC average). (**D**) Correlations between individual pattern similarity levels from (B) and a clinical summary score (so-called PANSS positive or negative scores; available only for patients with FEP), for MEM (red) and LZP (blue). (**E**) Comparison of the differences between correlations for positive and negative symptoms between drug conditions. The *P* value quantifies the significance of double dissociation (factors: drug and symptom dimension). All *P* values were assessed with two-sided permutation tests [corrected for spatial autocorrelation in (B) and (C)]. Error bars, 95% bootstrap confidence intervals.

We devised a second approach that enabled us to also assess the interindividual variability in the pattern similarities between their individual effects (compared to the group average HC maps) and the effects of the pharmacological interventions in healthy participants (Fig. 7). Here, we concatenated all parameters vertically, yielding a single column vector ( $u_{\text{ESP}}$ ), and for each drug effect ( $v_{\text{LZP}}$ and $v_{\text{MEM}}$ ). We computed $u_{\text{ESP}}$ as the difference between each individual parameter map from the CHR-P and FEP groups and the corresponding group average parameter maps for the HC group, yielding individual psychosis signatures for each participant from the clinical groups. As a common reference, $v_{\text{LZP}}$ and $v_{\text{MEM}}$ were computed by pooling the data of all healthy participants from the pharmacological study (group average drug effect). We then computed the similarity between individual psychosis signatures and the group average LZP or MEM effects (Fig. 7A).

As expected, this approach revealed substantial variability across both groups in terms of the similarity levels of individual psychosis signatures with the drug effects (Fig. 7, B and C). For both LZP and MEM reference patterns, we found statistically significant similarities (correlations) for several individuals from both clinical groups of opposite signs (Fig. 7, B and C; filled symbols above and below 0). For the MEM reference pattern, however, a comparable number of clinical participants exhibited positive or negative correlations. When pooling the individual correlations across the CHR-P and FEP groups (based on their high similarity; Fig. 3B), the group average correlation coefficient was indistinguishable from zero [*r*(1080) = −0.006 and *P* = 0.28; see Fig. 7, B and C for separate tests for each clinical group]. By contrast, the individual LZP similarities were mostly positive and yielding a significantly positive group average correlation, both pooled across CHR-P and FEP [*r*(1080) = 0.07 and *P* = 0.002], as well as for each clinical group individually (Fig. 7, B and C). The results indicate that individual psychosis signatures contained two components that were both spatially and neurochemically separable: one that was related to GABAergic inhibition and reasonably consistent across participants and another, less consistent one, that was related to glutamatergic excitation via NMDA receptors.

In the FEP group (but not the CHR-P group), positive and negative symptoms were assessed in terms of the Positive and Negative Symptoms Scale [PANSS; (*76*)]. We used these clinical scores to test if the individual differences in the expression of both components were related to individual clinical symptoms. To this end, we correlated the individual similarities for each drug pattern from Fig. 7B with either the aggregate scores of positive or negative symptoms (Materials and Methods). This revealed distinct symptom correlates of the spatial similarities to the LZP and MEM effects: MEM similarities were positively correlated with the strength negative symptoms [*r*(32)=0.43 and *P* = 0.01; Fig. 7D], while LZP similarities were positively correlated with the expression positive symptoms [*r*(32) = 0.49 and *P* = 0.004; Fig. 7D]. The differences in correlation with positive and negative scores for MEM and LZP, respectively, were highly significant (*P* = 0.004; Fig. 7E), demonstrating the specificity of these effects. In other words, patients with psychosis whose spatial patterns of alteration in cortical dynamics exhibited larger similarity to the changes by a boost of GABA-A transmission exhibited more severe positive symptoms, and patients with stronger similarity to changes induced by NMDA-R blockade exhibited more severe negative symptoms.

## DISCUSSION

The cerebral cortex spontaneously generates a rich repertoire of dynamical activity patterns, such as band-limited oscillations or aperiodic activity, which have been suggested as potential biomarkers for neuropsychiatric disorders (*12*, *22*, *23*, *34*, *38*, *40*). Recent work on the large-scale distribution of cortical microcircuit properties, such as cytoarchitectural type or receptor gene expression, identified substantial spatial heterogeneity in these parameters across cortical areas and cortical hierarchy as one overarching organizing principle (*24*, *46*). Here, we integrate these different bodies of work by leveraging the high spatiotemporal resolution and cortex-wide spatial coverage afforded by MEG source imaging (*43*) and combining the latter with state-of-the-art anatomical atlases (*55*) and pattern analyses.

We characterized large-scale cortical patterns of changes in several parameters of cortical dynamics in early stage psychosis (psychosis signatures) and related those to the patterns of receptor densities as well as to the patterns of changes in the same dynamics parameters induced by pharmacological manipulations of GABA-A or NMDA receptors (LZP versus MEM) in healthy individuals. For both clinical groups, FEP and CHR-P, we found a robust similarity between the respective group average psychosis signatures and the cortical distribution of GABA-A receptor densities, but no such relationship to NMDA receptor densities. Notably, in patients with FEP, we also observed a double dissociation in the correlation between individual symptoms (which were only assessed with clinical PANSS scores in that group) and the similarity of patients’ individual psychosis signatures with GABA-A or NMDA effect maps in healthy individuals: The individual similarity with the GABA-A (LZP) effect maps was related to the strength of their positive (not negative) symptoms, while the individual similarity of the NMDA (MEM) effect maps was related to the negative (not positive) symptoms. The observations that (i) the psychosis signatures were less strongly related to NMDA receptor densities, (ii) the pharmacologically defined “NMDA component” was less consistently expressed across different clinical participants, and (iii) that the “NMDA component” expression was unrelated to positive symptoms may be unexpected in light of the so-called “ketamine model” of psychosis (*14*, *77*–*79*). Even so, the large interindividual variability in the expression of that NMDA component we observed was systematically related to (negative) clinical symptomatology. Together, our results support the idea that both neurotransmitter systems, glutamate and GABA, are involved in the pathophysiology of psychotic disorders (*7*) but contribute in distinct ways to positive and negative symptoms.

### Neurotransmitter basis of psychosis signatures and symptoms

Considerable evidence implicates GABAergic cortical inhibition (*9*) and NMDA receptors (*12*, *13*, *34*, *80*) in psychotic disorders, in particular early stage psychosis (*81*). The observation of a lack of relationship between the patient average pattern of psychosis signature on cortical dynamics to either the NMDA receptor density levels may appear as inconsistent with a strong involvement of NMDA receptors. One possible explanation for this lack of correlation for our analyses of resting-state activity is that the alterations in circuit dynamics due to NMDA hypofunction in psychosis are more strongly and consistently expressed during effortful cognitive processes that recruit synaptic reverberation via NMDA receptors (*13*, *26*, *80*) [but see (*81*)]. This possibility should be tested in future work applying a similar approach to MEG obtained during performance of tasks involving working memory or evidence accumulation for decision-making.

The interindividual differences in similarity levels to NMDA versus GABA-A neurotransmitter manipulations have implications for individualized approaches in psychiatry research and clinical practice. Our approach affords mapping the MEG signature of each individual in the clinical groups on two axes of similarity (GABA-A and NMDA effects), in an objective and quantitative manner. The fact that these axes are obtained by comparison with controlled manipulations of two well-defined receptor types that have been implicated in the pathogenesis of psychosis implies that the resulting values are mechanistically grounded. Our finding that these interindividual differences also explained substantial fractions of interindividual variability in distinct symptom classes means that the resulting values are also clinically meaningful. The approach introduced here may, therefore, provide a useful basis for the stratification of patient cohorts in future research of psychosis or related neuropsychiatric disorders.

We also encourage future studies quantifying positive versus negative symptoms in CHR-P individuals, to test if the double dissociation in the correlation between individual symptoms and similarity of psychosis signatures with NMDA versus GABA-A effect maps that we observed for FEP is also present before the full clinical manifestation of psychosis.

### Local effects versus spatial patterns

We found only weak local differences in cortical dynamics parameters under either pharmacological manipulation of GABA-A and NMDA receptors or in clinical groups compared to HCs. The strengths of pharmacological effects will scale with the drug doses, which were low in our study, to minimize secondary and side effects and maximize specificity of effects for the receptors with highest affinity. However, the local expression strengths of the psychosis signatures are given by the nature of the disorder and therefore unlikely to change substantially between different clinical samples (they may be aggravated in a later stage of psychosis). This highlights the importance of our approach focusing on spatial pattern similarity, and specifically the similarity of the difference patterns (clinical participants versus controls) for picking up locally subtle, but spatially highly consistent, reproducible, and clinically relevant features of large-scale cortical dynamics. The observations that the large-scale patterns of these psychosis signatures and drug effects were (i) highly reproducible across independent datasets and (ii) reflected an important synaptic feature of brain organization, the cortex-wide distribution of GABA-A receptor densities, establish that these patterns are genuine and meaningful.

Our approach was inspired by insights from multivariate pattern analyses of mental representations in neuroimaging (*82*, *83*). Conventional mass-univariate approaches were designed to detect differences in the activity of individual sources (e.g., functional MRI voxels) between conditions. Such local differences, while robust for coarsely defined conditions (e.g., high versus low cognitive load), are often too subtle for discriminating between conditions differing only in specific stimulus or cognitive variables (*82*, *83*). Detection of small effects at the level of single sources is limited by the multiple-comparisons problem associated with mass-univariate statistical tests (*83*). By combining the information contained in many such small, but systematic, local effects, multivariate pattern analyses boost sensitivity (*84*, *85*). Spatial pattern correlations similar to ours, just operating at the level of within-area voxel patterns, are at the heart of state-of-the-art representational similarity analyses (*86*). While guided by the same methodological insights, our current approach is designed for a different purpose: identifying and explicitly relating large-scale patterns of changes in cortical dynamics induced by pharmacological interventions or brain disorders.

We here correlated one-dimensional pattern vectors. This approach was simple, consistent with previous work quantifying the large-scale organization of the cerebral cortex (*46*). It yielded several significant effects, including correlations on the order of 0.8 to 0.85, highlighting its sensitivity. However, a limitation of the approach was its lack of sensitivity to the two-dimensional arrangement of areas on the cortical surface. More sophisticated spatial pattern analyses in future studies, for example, operating on flat maps of the cortical sheet, may further advance our current insights.

### Relationship to previous work on cortical dynamics

Several previous studies in schizophrenia have investigated the EEG or MEG markers that were part of our six-parameter assay, with mixed results (*15*–*21*, *65*, *73*, *87*). These studies have commonly focused on a single parameter of cortical population activity (e.g., aperiodic exponent), or a small subset of them. Two EEG studies reported an increase of the aperiodic exponent (i.e., steeper exponent) in patients with schizophrenia, across many scalp electrodes during a cognitive task (*16*, *73*). Another EEG study found no significant differences in the aperiodic exponent between patients with FEP and HCs (*17*). One previous MEG sensor-level analysis of the current clinical dataset found the Hurst exponent, overall and for a subset of parietal sensors, to be similar across CHR-P, FEP, and HC groups (*15*). Several E/MEG studies have shown that the power of spontaneous alpha-band activity is decreased in schizophrenia (*18*–*21*), however, without decomposing the power spectra into periodic and aperiodic components, which may confound the estimates of band-limited power (*65*). One study has also reported a slower alpha peak frequency in patients with schizophrenia (*87*). None of these previous studies has comprehensively quantified the cortex-wide patterns of psychosis-related alternations of multiple dynamic parameters in source space, as we did here. This pattern analysis was essential for identifying the relationship between the neurophysiological psychosis signatures to neurotransmitter systems and symptoms in the current work.

A large body of work has demonstrated psychosis-related aberrations of neuronal responses in the gamma band (*22*, *62*), which are, in turn, generated by excitatory-inhibitory synaptic interactions within cortical microcircuits (*44*, *68*, *69*, *88*). We here focused on alpha- rather than gamma-band oscillations for the quantification of periodic components of local cortical dynamics. This choice was pragmatic, because our approach relied on across-area patterns, and alpha peaks were more consistently detected across cortical areas in the resting-state data analyzed here. Under conditions of strong external (stimulus and/or task) drive of local cortical circuits, gamma-band responses will be more prominent (*44*), and future applications of our approach to such data may also use gamma-band oscillations. That said, the alpha-band oscillations used here are likely to provide relevant information complementary to the information carried by parameters describing the aperiodic activity components, for example, due to a contribution of thalamocortical interactions to the generation of cortical alpha-band oscillations (*89*).

We replicated the observation from previous work (*39*, *51*, *90*, *91*) that the spatial patterns of cortical dynamics parameters followed the anatomical hierarchy (*46*), which, in turn, has been identified as an important organizing principle that accounts for a large fraction of variation in gene expression and microcircuit architecture across the cortex (*24*, *46*). We extended this finding in two important ways. First, we show that this relationship is preserved in early stage psychosis as well as under pharmacological manipulations of NMDA and GABA-A receptors. Second, we also show that the psychosis signatures were only weakly associated with anatomical hierarchy but strongly associated with GABA-A receptor density levels as well as the maps of pharmacological manipulation effects. This points to the presence of other principles of large-scale brain organization beyond anatomical hierarchy (*92*, *93*), which may be important for understanding the pathophysiology of psychotic disorders.

### Nature of the large-scale psychosis signature

Does the multiparametric psychosis signature that we identified directly generate the symptoms of psychosis? Emergent features of neural mass activity, such as oscillations (*44*) or long-range temporal correlations (*40*), may not be directly used in the neural computations underlying behavior (*94*). The same will hold for the relationship between changes of such MEG markers on the one hand and disorders of cognition on the other hand. However, at least a subset of the parameters we assessed here are likely produced by the microscopic features of cortical circuits that also give rise to psychosis, for example, the strength of recurrent excitation and/or inhibition in the network, an assumption supported by the here-established link to the neurotransmitter systems involved intracortical recurrent excitation and inhibition. In other words, changes of these circuit properties are likely to be a common cause of both, MEG markers and clinical symptoms. This highlights the importance of our mechanistically informed selection of the parameters of the current assay: features of neural mass action that are more remote from the intracortical circuit mechanisms generating psychosis [e.g., phase-locked components of stimulus responses; (*44*)] may be less useful for identifying psychosis signatures that relate to large-scale cortical neurotransmitter distribution and function. An important avenue for further research will be to fit biophysical models to convert the current multiparameter maps into maps of the underlying (microscopic) circuit parameters (*41*), the alterations of which directly generate the symptoms of psychosis (*41*).

To conclude, our results indicate that the large-scale neurophysiological signatures of early stage psychosis (in part) reflect alterations in GABA-A or NMDA receptor functions and relate to individual symptomatology. Our results corroborate the notion that the pathophysiology of psychosis is fundamentally distributed across the brain and establish a mechanistic foundation for the stratification of early stage psychosis cohorts and biomarker development.

## MATERIALS AND METHODS

We analyzed resting-state MEG datasets collected in two separate studies: (i) a study (*60*) comparing CHR-P participants, FEPs versus HCs; and (ii) a pharmacological study (*61*) in young and healthy participants with double-blind and placebo-controlled manipulations of GABA-A or NMDA receptors, respectively.

### Participants and informed consent

#### Clinical dataset

Three groups of participants were recruited to the study. One group included participants who met CHR-P criteria (*n* = 117, mean age of 22 years, range of 16 to 24 years, 83 females), based on the Comprehensive Assessment of At-Risk Mental State interview (CAARMS) (*95*) and the Cognitive-Perspective Basic Symptoms (COGDIS/COPPER) item of the Schizophrenia Proneness Instrument (SPI-A) (*96*). Exclusion criteria included diagnosis of Axis I psychotic disorders, including affective psychosis, as evaluated by the Structured Clinical Interview for the Diagnostic and Statistical Manual of Mental Disorders–IV (SCID). In addition, an FEP group (*n* = 32, mean age of 24 years, range of 18 to 34 years, 12 females) was recruited, for which clinical symptoms were assessed in terms of the PANSS (*76*).

The control group for both above clinical groups was a sample of age- and gender-matched HCs (*n* = 45, mean age of 23 years, range of 18 to 32 years, 31 females), who were screened for psychopathology using the SCID or the MINI-SCIS interview. The HC group was matched by age to the two other group (HC versus FEP and CHR-P combined: *P* = 0.48, two-sided permutation test), but not by years of education (HC: 16.8 years; CHR-P and FEP combined: 15.1 years; *P* = 0.002, two-sided permutation test). There was a similar percentage of females in the HC and in the combined clinical sample (68.8 and 63%, respectively; *P* = 0.47; χ^2^ test). The three groups of participants were recruited as part of the Youth Mental Health Risk and Resilience study [see (*60*) for more information about participants’ recruitment, exclusion criteria, and experimental procedures], which was approved by the NHS Research Ethical Committee Glasgow (reference number: 14/WS/0099). All participants provided written informed consent and received remuneration of 36£. We excluded three participants (all from the CHR-P group) due to excessive artifacts in MEG signals.

#### Pharmacological dataset

We recruited 23 healthy participants (mean age of 28 years, range of 21 to 40 years, 9 females) to the study. Exclusion criteria included the following: impaired vision or hearing; illegal drug use; regular medication intake; consumption of more than 15 units of alcohol per week; known hypersensitivity to MEM or LZP; past or current psychiatric or neurological diagnosis; cardiovascular, liver, kidney, or metabolic diseases; pregnancy; and nonremovable metallic parts (e.g., insulin pump and retainer). We excluded one participant after detecting excessive metal artifacts in the MEG signal, and two participants dropped out before completing all experimental sessions. Participants received remuneration of 15€ for the training session, 100€ for each MEG session, 150€ as a completion bonus, 10€ for MRI session, and a performance-dependent bonus of a maximum of 150€. The study was approved by the ethics committee of the Hamburg Medical Association (reference number: 2021-100608-BO-ff), and all participants provided written informed consent.

### Experimental design

#### Clinical dataset

The three groups of participants (CHR-P, FEP, and matched controls) completed one MEG session (~3.5 hours long) followed by one magnetic resonance spectroscopy/MRI session (~2.5 hours). Among other tasks in the MEG (*60*, *64*), participants completed a 5-min block of resting state recording with eyes open (fixation of a central cross on an otherwise gray screen). Participants were instructed to keep fixation at a plus sign in the center of a gray screen and keep a blank state of mind as well as possible (i.e., do not think of anything in particular and if a thought comes up, do not follow up on it).

#### Pharmacological dataset

Participants completed one training session (outside the MEG) followed by six MEG sessions, interleaved by at least 1 week (Table 2). Each MEG session started with the intake of a pill (LZP, MEM, or placebo; see below), followed by a 150-min waiting period before MEG recording. In the MEG, participants performed several different tasks, lasting approximately 2.5 hours. This included a 10-min block of resting state recording with eyes open (fixation of a central cross on an otherwise gray screen). This resting state recoding took place at the end of the session, around 4.5 hours after drug intake. During the block, participants seated quietly with their eyes open and asked to maintain fixation at a centrally presented black cross against a gray background throughout the block.

**Table 2.** Time course of pharmacological design. Each row shows the order of pharmacological conditions per participants. Numbers in parenthesis are times (in weeks) from last session. PLA, placebo; LZP, lorazepam; MEM, memantine.

Participant ID	Sess 1	Sess 2	Sess 3	Sess 4	Sess 5	Sess 6
**S01**	MEM	LZP (5)	LZP (1)	PLA (2)	MEM (1)	PLA (5)
**S02**	MEM	LZP (5)	MEM (1)	PLA (2)	LZP (8)	PLA (1)
**S03**	LZP	MEM (1)	PLA (1)	LZP (1)	MEM (1)	PLA (1)
**S04**	PLA	LZP (5)	LZP (1)	MEM (1)	PLA (1)	MEM (1)
**S05**	PLA	LZP (1)	MEM (1)	LZP (1)	PLA (3)	MEM (1)
**S06**	LZP	MEM (1)	PLA (1)	MEM (1)	LZP (1)	PLA (1)
**S07**	MEM	LZP (4)	LZP (1)	PLA (1)	PLA (1)	MEM (1)
**S08**	LZP	PLA (7)	PLA (1)	MEM (5)	LZP (1)	MEM (1)
**S09**	LZP	PLA (5)	PLA (1)	MEM (1)	MEM (1)	LZP (1)
**S10**	MEM	LZP (5)	PLA (1)	LZP (1)	MEM (1)	PLA (1)
**S11**	LZP	MEM (6)	MEM (1)	PLA (1)	PLA (1)	LZP (1)
**S12**	PLA	LZP (5)	MEM (1)	PLA (1)	MEM (1)	LZP (1)
**S13**	MEM	PLA (1)	LZP (1)	PLA (1)	MEM (3)	LZP (1)
**S14**	PLA	MEM (1)	LZP (2)	PLA (1)	LZP (2)	MEM (1)
**S15**	LZP	MEM (1)	PLA (2)	LZP (1)	PLA (3)	MEM (1)
**S16**	MEM	Missing data	PLA (2)	LZP (1)	MEM (3)	LZP (1)
**S17**	MEM	LZP (1)	LZP (6)	PLA (1)	MEM (1)	PLA (2)
**S18**	MEM	PLA (1)	LZP (1)	MEM (1)	LZP (1)	PLA (2)
**S19**	PLA	MEM (1)	PLA (1)	LZP (1)	LZP (3)	MEM (1)
**S20**	PLA	MEM (3)	MEM (1)	PLA (1)	LZP (1)	LZP (1)

We orally administered LZP or MEM, in a double-blind, placebo-controlled, and a crossover design. Each drug was given twice, in two different experimental sessions, in addition to two placebo sessions. LZP is a GABA-A receptor agonist that boosts GABAergic neurotransmitters. MEM is a NMDA receptor antagonist that decreases glutamatergic neurotransmitter. We choose subclinically dosage for the drugs: 1 mg for LZP (common clinical daily use between 0.5 and 2 mg) and 15 mg for MEM (common clinical dose, 20 mg). Peak plasma concentration for LZP is 2 to 3 hours and for MEM is 3 to 8 hours. Thus, we administered the drugs 3 hours before MEG session started (2.5-hour waiting period plus 30-min preparation time before MEG recording), to maximize plasma concentration levels. To allow plasma concentration level to return to normal, experimental sessions were interleaved by at least 1 week (plasma half-life of LZP is ~13 hours and that of MEM is 60 to 70 hours; Table 2).

We here used MEM instead of ketamine, an NMDA receptor antagonist with a mechanism of action resembling the one of MEM (*97*). This was for two reasons: (i) the oral administration of MEM matches the one of LZP, improving the blinding in our within-participants intervention and (ii) low dosage help to minimize nonspecific secondary effects (*97*). Ketamine has strong effects on the aperiodic exponent at high (*98*) but not low (*99*) dosage. At subanesthetic levels, it can produce symptoms in healthy participants that resemble symptoms of psychosis (*14*, *77*–*79*). MEM effects on cortical dynamics (specifically, aperiodic exponent) also increases with dosage (*16*).

### Data acquisition

#### Clinical data

##### MEG

MEG data were collected at the Center for Cognitive Neuroscience at University of Glasgow, using 248 channels 4D-BTI system (4D-Neuroimaging, San Diego), at a sampling rate of 1017.25 Hz. Online low-pass filter was applied at 400 Hz.

##### MRI

T1-weighted data were obtained from 178 of 191 participants included in the final analyses reported here, using a 3T Siemens Magnetom Trio MRI scanner at the Center for Cognitive Neuroscience at University of Glasgow with the following parameters: 192 slices, voxel size = 1 × 1 × 1 mm^3^, field of view (FoV) = 256 × 256 × 176 mm^3^, repetition time (TR) = 2250 ms, echo time (TE) = 2.6 ms, and flip angle = 9°.

#### Pharmacological data

##### MEG

MEG data were collected at the Department of Neurophysiology at University Medical Center Hamburg-Eppendorf using a CTF MEG system with 275 gradiometers and a sampling rate of 1200 Hz. Participants were seated in a dark and magnetically shielded room and were asked to minimize head movements. We monitored and recorded head positions continuously, using three fiducial coils, placed on participants’ ear canals and nasal bridge. In addition, we recorded ECG and vertical and horizontal electrooculogram (EOG) using Ag-AgCl bipolar electrodes with a sampling rate of 1200 Hz. Eye movements were recorded using EyeLink 1000 system, with a sampling rate of 1000 Hz. We calibrated gaze positions at the beginning of the block.

##### MRI

T1-weighted images were obtained from all participants using a 3T Siemens Magnetom Trio MRI scanner (Siemens Medical Systems, Erlangen, Germany) and the following parameters: voxel size = 1 × 1 × 1 mm^3^, TR = 2300 ms, TE = 2.98 ms, FoV = 256 mm, slice thickness = 1 mm, TI = 1100 ms, and flip angle = 9°.

### MEG data analysis

We analyzed both datasets (pharmacological and clinical) using the same procedures, with several adjustments due to differences in the acquisition protocols. Unless stated otherwise, the following refers to both datasets. We analyzed MEG data using customized code from Fieldtrip toolbox (*100*) in MATLAB (MathWorks) and MNE (*101*) and pymeg (https://github.com/DonnerLab/pymeg) toolboxes in Python. We preprocessed MRI scans using FreeSurfer.

#### Preprocessing

Preprocessing of MEG data included three steps. The first step detected MEG artifacts to be rejected from the signal before applying independent component analysis (ICA; second step), to avoid extreme values affecting the ICA. The third step rejected data segments containing previously identified artifacts and removed ICA components from the remaining continuous data. Each step applied different high-pass filters with cutoffs adapted the respective purpose. Hence, each step started from the raw unfiltered data.

The first step identified segments containing head movements, muscle contractions, squid jumps, or metal artifacts. Continuous MEG and external electrodes (ECG) were down-sampled to 400 Hz, and band-pass filtered to remove powerline noise at 50, 100, and 150 Hz (two pass Butterworth filter; band stop frequencies, ±1 Hz around each frequency). We detected muscle artifacts by band-pass filtering MEG signals between 110 to 140 Hz and *z* scoring; values exceeding *z* = 10 were labeled as muscle artifacts. Head movements (only for pharmacological dataset; continuous monitoring of head movements was not done in the clinical dataset) were detected as deviation of any coil more than 6 mm from the template head position. Squid jumps were detected by fitting a line to the log power spectrum of individual artificial trials (7 s long) and detecting outliers of its intercept. Last, metal artifacts (e.g., cars passing near the building) were detected as samples exceeding a threshold of ±5 pT.

In a second step, the raw MEG data were high-pass filtered at 1 Hz, and segments containing squid jumps, head movements, and metal artifacts were rejected, to avoid extreme values during ICA. We then applied ICA using the infomax algorithm as implemented in MATLAB and manually identified components containing eye movements (i.e., blinks and saccades) and heartbeats.

In a third step, we resampled the raw data and removed power line noise, using the same parameters described above for the first step, high-pass filtered the data at 0.1 Hz (Butterworth filter) and removed previously identified ICA components or rejected data segments previously identified as containing artifacts (muscle contractions, head movements, metal artifacts, and squid jumps), respectively. We did not reject segments containing eye movement–related activity (blinks or saccades), but rather removed ICA components containing these artifacts. This was done to match the preprocessing steps between the two datasets, where the clinical dataset did not include any monitoring of eye movements (i.e., neither EOG nor eye-tracking data).

In total, our procedure led to the rejection of 13% of data segments in the clinical dataset and 26% of data segments in the pharmacological dataset, resulting in time series with duration of 262 s ± 36 s (mean and SD across participants) or 437 s ± 56 s, respectively.

#### Source reconstruction

We used linearly constrained minimum variance (LCMV) beamforming to project broadband sensor-level signals into the cortical source space. We constructed a three-layer head model (inner skull, outer skull, and skin) for each participant based on their individual MRI scan. In cases where an MRI scan was missing, we used the FreeSurfer fsaverage to reconstruct a template head model. We aligned the head model to MEG data by computing a transformation matrix, using the mean coils positions across the resting state block (for pharmacological dataset), or a fixed coils positions measured at the beginning of the block (for clinical dataset) and the corresponding locations of fiducial coils on the individual head model. We then used the individual MRI scan and FreeSurfer to reconstruct cortical surfaces and parcellate the cortex into 360 parcels, using the anatomical atlas from Glasser *et al.* (*55*). On the basis of the head model, we generated a forward model (“leadfields”) for the computation of LCMV filters, which was confined to the cortical sheet (4098 vertices per hemisphere, recursively subdivided octahedron). For each vertex, a set of LCMV filters (one per cardinal orientation) were computed by combining the leadfield matrices of that vertex with a covariance matrix of the sensor-level signal. For each vertex, we chose the source orientation with the maximum source power using LCMV filters. We projected the time series through the LCMV filters into source space and collapsed the data across all vertices within each parcel. This resulted in 360 time series, one for each parcel, of source-level broadband data.

#### Quantification of local cortical circuit dynamics

We estimated power spectral density (PSD) by dividing the time series of each parcel into 10-s epochs, with 50% overlap, transforming each epoch into the frequency domain using the Fourier transform, and averaging the absolute values of Fourier coefficients across all epochs. This resulted in PSDs with a frequency resolution of 0.1 Hz. We smoothed the PSD at 50, 100, and 150 Hz using linear interpolation (±2 Hz around each frequency) since activity at those frequencies was filtered during preprocessing. We fitted the FOOOF model (*65*) to each PSD in a range between 1 and 65 Hz, to avoid the noise floor at higher frequencies (*102*), and included a knee parameter, based on a visual inspection of the PSDs. If the algorithm failed to fit the model or returned a negative knee frequency, we re-fitted the model without the knee parameter. Additional fitting parameters were as follows: peak threshold, 2 SDs; and peak limit, [2 16] Hz. We extracted from the model the aperiodic exponent, knee frequency (if fitted, otherwise a NaN was assigned), alpha power, and its peak frequency. Alpha power was computed as the difference in power between the fitted periodic and aperiodic components, at its peak frequency, and reported in arbitrary units. We noticed that in some cases, multiple peaks within the frequency range from 7 to 13 Hz were detected by the algorithm. To identify alpha peak more accurately, we constrained the range of searching by first fitting the model to the mean PSD across V1 and early visual cortex (V2 to V4), where alpha activity is the strongest, and extracted a single alpha peak frequency and its bandwidth, after visually verifying it is indeed in the alpha range. We used this frequency and its bandwidth (±2 Hz) to constrain the range of searching for alpha peaks in all other parcels. If still more than one peak was identified within this range, we chose the one with the highest power. In rare cases, no alpha peak could be detected from the visual cortex. In such events, we selected the peak with the highest power within the traditional alpha range of 7 to 13 Hz. If no peaks were detected whatsoever, we treated those as missing values (i.e., NaNs) for all further analysis. We computed the AUC from the aperiodic fit of the PSD, by summing the power between 1 to 65 Hz. The Hurst exponent of spontaneous fluctuations of amplitude envelopes in the alpha-band oscillations was computed as in (*40*). In brief, we used detrended fluctuation analysis, as implemented in NBT toolbox (Neurophysiological Biomarker Toolbox: https://github.com/NBT-Analytics/NBTpublic), with the following parameters: filter range, 8 to 13 Hz (order 100 finite impulse response filter); calculating range, 1 to 120 s (50% overlap); and fitting range, 3 to 50 s. The six dynamics parameters extracted here were not correlated by construction. Such trivial correlations exist, for example, for the exponent and intercept of the aperiodic component. Our parameter selection ensured that any (across-area) correlations between parameters identified in the data were meaningful and interpretable in terms of underlying mechanism or shared organizational principles (such as anatomical hierarchy).

### Receptor density maps

We used the publicly available volumetric PET images from (*53*). We used the group-averaged PET images of two neurotransmitter receptors: GABA-A and NMDA. PET images were registered into the MNI template and then parceled into 360 parcels according to the above-mentioned parcellation (*55*) and then *z* scored across parcels.

### Polynomial regression for projected maps

We projected the difference maps of CHR-P versus HC or FEP versus HC on four axes in the anatomical MNI standard space: *x* (left-right), *y* (anterior-posterior), *z* (inferior-superior), or the diagonal between *y* and *z* axes (i.e., *y* + *z*). We then fitted a sequence of polynomial regression models

$$y=\sum_{i=1}^{n+1}p_{i}x^{n+1-i}$$ (1)

where *n* was the model order (*n* ≥ 1), and $p_{i}$ are model coefficients. We used *R*^2^ to estimate model fit and tested (using *F* test for nested models), if *R*^2^ improved when *n* increased. We stopped when *n* + 1 (full model) did not significantly improve compared to *n* (nested model). In all cases, either a first-order or a second-order polynomial model best explained the data, whereby first-order models indicated a monotonic and linear change along the respective axis, and a second-order model indicated a quadratic fit, and a nonmonotonic change along the axis.

### Canonical correlation analysis

We used CCA to relate the psychosis signature on the cortical patterns of the six dynamics parameters to the matching drug effect on them. In the clinical dataset, we first *z* scored the cortical spatial pattern of each of the six parameters, computed the mean across both clinical groups (i.e., pooled across CHR-P and FEP), and lastly calculated the psychosis signature by subtracting from the mean clinical participants the mean across HCs. Similarly, in the pharmacological dataset, we *z* scored each parameter for each participant and drug condition, and then calculated the drug effect as the mean across participants of the difference between each drug condition and the placebo condition. We then horizontally concatenated the group average spatial patterns of all six parameters into two 180 × 6 matrices, which we denoted *X* for the psychosis signature and *Y* for the drug effect. We performed two separate CCA’s for the LZP and MEM effects, respectively, so below *Y* refers indiscriminately to LZP or MEM effects, since the computation was identical for both effects.

CCA decomposed the relationship between the two multivariate datasets (i.e., the pair of matrices *X* and *Y*) into orthogonal sets of latent variables according to

$$Z_{x}={\mathrm{XW}}_{x},Z_{y}={\mathrm{XW}}_{y}$$ (2)

where $W_{x}$ and $W_{y}$ were 6 × 6 matrices of linear weights, which X and Y were projected onto, resulting in 180 × 6 matrices $Z_{x}$ and $Z_{y}$ , the columns of which corresponded to the six latent (so-called “canonical”) variables. The weights were chosen so as to maximize the correlations between each pair of canonical variables (i.e., corresponding columns of $Z_{x,},Z_{y}$ ). These pairs of columns of $Z_{x}$ and $Z_{Y}\;$were ordered by the magnitude of their respective correlations, such that the first pair had the largest correlation, and each subsequent pair had progressively smaller correlations, while being orthogonal to all other pairs. The weight matrices ( $W_{x},W_{y}$ ) satisfying these constraints were found by first applying a *QR* decomposition to *X* and *Y*

$$X=Q_{x}R_{x,}\;Y=Q_{y}R_{y}$$ (3)

where *Q* were 180 × 6 orthogonal matrices (columns were orthogonal unit vectors) of dimensionality, and *R* were 6 × 6 upper triangular matrices. The term $Q_{x}^{T}Q_{y}$ was subjected to singular value decomposition

$$Q_{x}^{T}Q_{y}=\mathrm{US}V^{T}$$ (4)

where *U* and *V* were 6 × 6 orthogonal matrices, and *S* was a 6 × 6 diagonal matrix containing the canonical correlation coefficients in ascending order, such that diag(S)=R , where *R* was a vector of canonical correlation coefficients. The weight matrices were computed as

$$W_{x}=R_{x}^{-1}U\;\text{and}\;W_{y}=R_{y}^{-1}V$$ (5)

The amount of covariance explained by each set of canonical variables equaled *R*^2^.

To quantify the relative contribution of each dynamics parameter to the canonical correlation for the first canonical variables (first columns of $Z_{x}$ and $Z_{y}$ ), we computed the so-called “loadings” as the Pearson’s correlation coefficients between these canonical variables and the original data (psychosis signature or drug effect) matrices. For each CCA (i.e., the LZP and the MEM analysis), this yielded a pair of 1 × 6 vectors, with one entry per dynamics parameter, for the contributions of parameters in drug effect and in psychosis signature, which were tested further for their reliability as described below.

To demonstrate the reproducibility of the CCA results, we repeated the analysis for each clinical group (i.e., CHR-P and FEP) and for each pair of the repeated sessions (S) of a pharmacological condition (e.g., LZP S1 and LZP S2). This resulted in a total of four pairs for each drug condition: session S1-CHR-P, S2-CHR-P, S1-FEP, and S2-FEP. For each combination, we computed the canonical variables matrices (e.g., $Z_{x\left(\text{CHR},S1\right)}$ and $Z_{y\left(\text{CHR},S1\right)}$ ) and corresponding loading vectors, and quantified the similarity in the resulting (spatial or parameter) patterns across sessions and clinical groups by calculating the correlation between pairs of canonical variables (e.g., $Z_{x\left(\text{CHR},S1\right)}\;\text{and}\;Z_{x\left(\text{CHR},S2\right)}$ ) or between pairs of corresponding loading vectors, across all combinations. This was done separately for variables or loadings associated with psychosis signatures and drug effects.

### Individual correlations of spatial patterns of effects

We tested the similarity between the effect of MEM or LZP on the spatial patterns of cortical dynamics parameters to the individual effect of psychosis. Parameters were *z* scored as described in the section above, and the drug effect was computed in the same way. The individual psychosis signature, however, was computed as the difference between each clinical participant and the mean across all participants in the HC group. We then concatenated all parameters vertically into a single column vector for each clinical participant ( $u_{\text{ESP},i};i=1\ldots N$ clinical participants), and for each drug effect ( $v_{\text{LZP}}$ and $v_{\text{MEM}}$ ). The vectors u and v had the same length of 6 (parameters) times 180 (parcels). To assess the spatial similarity between group average maps of drug effect and individual maps of psychosis signature (map for each clinical participant versus average map for HC group), we computed the correlation coefficient between each clinical participant map and each drug effect map (LZP or MEM) as $R_{\text{LZP},i}=\text{corr}\left(u_{\text{ESP},i},v_{\text{LZP}}\right);i=1\ldots N$ clinical participants (same for MEM).

### Pattern classification

We trained multivariate pattern classifiers to discriminate between clinical groups (CHR-P or FEP versus HC). We used support vector machines for all results reported in the main paper but achieved similar performance for another nonlinear classifier (random forest) and slightly worse performance for a simple linear classifier (logistic regression). To ensure balanced classes, we first randomly sampled the larger group (CHR-P, for CHR-P versus HC; and HC, for FEP versus HC). Next, the multivariate patterns of parameters (180 areas times 6 parameters; or a subset of the latter) of each individual participant were submitted to principal components analysis for dimensionality reduction. The top principal components together explaining 95% of the total variance in the data were used (typically around 20 components). We used an SVM classifier (using the MATLAB function fitcsvm) with the “rbf” kernel, for which we optimized two hyperparameters: BoxConstraint and KernelScale. We used the parameters maximizing classification accuracy to train and test the model using leave-one-out cross-validation. We quantified the model performance as the mean classification accuracy across all folds. We repeated the above procedure 200 times, each time randomly subsampling the larger group. The classification accuracies reported in Table 1 were the mean accuracies across all repeats. We also quantified and reported the confusion matrix of each classification problem (fig. 6), and computed the F1 score as

$$F1\;\text{score}=\frac{2\text{TP}}{2\text{TP}+\text{FP}+\text{FN}}$$ (6)

where TP was the true positive rate, FP was false positive, and FN was false negative. To rigorously quantify the classifier ability to separate between the two classes, we also computed the ROC curve, by taking the continuous classifier outputs (i.e., probabilities of the predicted samples to be CHR-P or FEP), and then computed the area under the ROC curve (AUC). AUC = 1 indicates a perfect separation, while AUC = 0.5 indicates no separation between classes. We obtained 66% confidence intervals of AUC values as the lowest 17 and highest 83rd percentiles of the AUC distribution across the 200 random subsamplings. We tested the statistical significance of classification accuracy using permutation testing: We randomly shuffled the label of the classes 500 times, for each shuffle again trained and tested the model using leave-one-out cross-validation and computed the mean classification accuracy across all folds. We compiled the resulting accuracy values in a null distribution concatenated across all repeats. *P* values were obtained as the probabilities of observing the accuracy from the true (nonshuffled) data under this null distribution.

### Statistical analyses

We computed the correlations between cortical patterns maps (e.g., psychosis signature or drug effect on dynamics parameters, receptor densities, and cortical hierarchy) using Spearman’s rank correlation. To estimate the statistical significance of correlations across parcels, we used spatial autocorrelation-preserving permutation tests. We generated null models using the spin rotation method (*103*, *104*) where the cortical surface is projected into a sphere and then randomly rotated, generating permuted cortical surfaces with preserved spatial autocorrelations. We used the spherical projection of the FreeSurfer fsaverage surface for the atlas (https://github.com/rudyvdbrink/Surface_projection) to assign each parcel with the coordinates of the vertex that is closest to the center of mass of the parcel. We then randomly rotated the coordinates and reassigned each original parcel with the value of the closest rotated parcels. We repeated this procedure 10,000 times. For parcels of which the medial wall was the closest, we assigned the value of the next closest parcel. Note that we implemented the sphere rotation on the parcel-level surface, to avoid repeated values within a single permutation which often occur when re-parceling vertex-resolution surfaces (*105*). The same procedure was applied to assess the statistical significance of CCA (Fig. 6) and of each correlation coefficient from Fig. 7. We applied the same rotation angle for all parameters within a single permutation (but randomly across permutations) before vertically or horizontally concatenating them. We computed the confidence interval using bootstrapping: We first sampled the data with replacement 10,000 times, and each time computed the relevant statistic (e.g., correlation coefficient and canonical correlation). We then calculated the lowest 2.5 and highest 97.5th percentiles of the resulting distribution, which we considered as the confidence interval.

## Supplementary Materials

### This PDF file includes:

Figs. S1 to S8 References
