# Supplementary material for: Large-scale maps of altered cortical dynamics in early-stage psychosis are related to GABAergic and glutamatergic neurotransmission
Source: Sci Adv. 2025 Aug 13;11(33):eads0400. doi: 10.1126/sciadv.ads0400 (PMC12346269; doi:10.1126/sciadv.ads0400)
Supplement: Supplementary file 1 — Figs. S1 to S8 References [file sciadv.ads0400_sm.pdf]

Science Advances
AAAS

Supplementary Materials for

Large-scale maps of altered cortical dynamics in early-stage psychosis are related to GABAergic and glutamatergic neurotransmission

Ayelet Arazi *et al.*

Corresponding author: Ayelet Arazi, a.arazi@uke.de; Tobias H. Donner, t.donner@uke.de

Sci. Adv. **11**, eads0400 (2025)
DOI: 10.1126/sciadv.ads0400

This PDF file includes:

Figs. S1 to S8
References

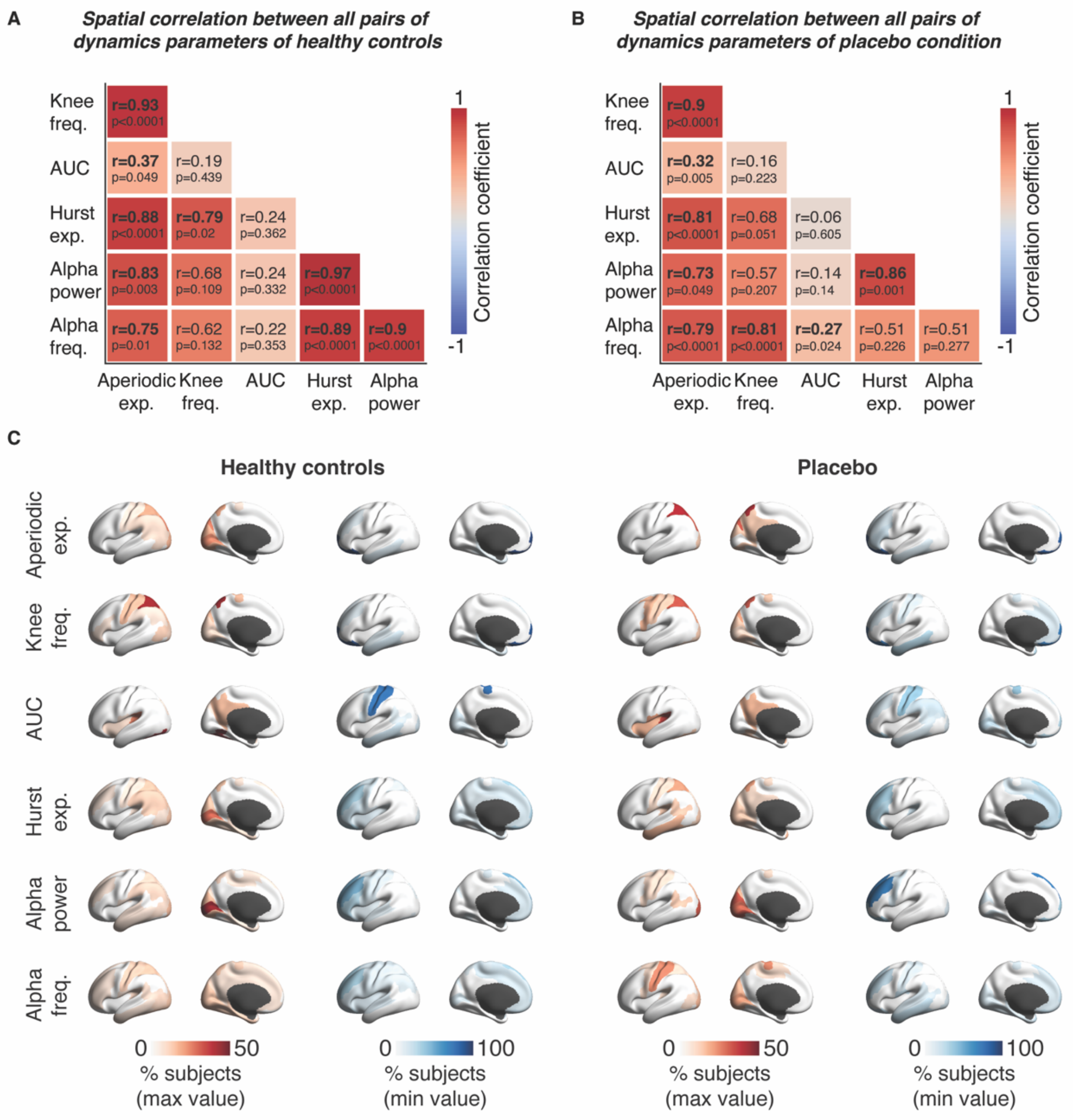

Figure S1: Quantification of the spatial correlation structure of spontaneous cortical dynamics.

(A, B) Matrices of the across-area correlations between all pairs of parameters within each sample. In all panels, the statistical significance of all Spearman correlation coefficients was tested using spatial autocorrelation-preserving permutation tests. **(C)** Percentage of subjects presenting local maximum (left, red) or minimum (right, blue) peaks across all parcels, grouped into 22 ROIs (*55*).

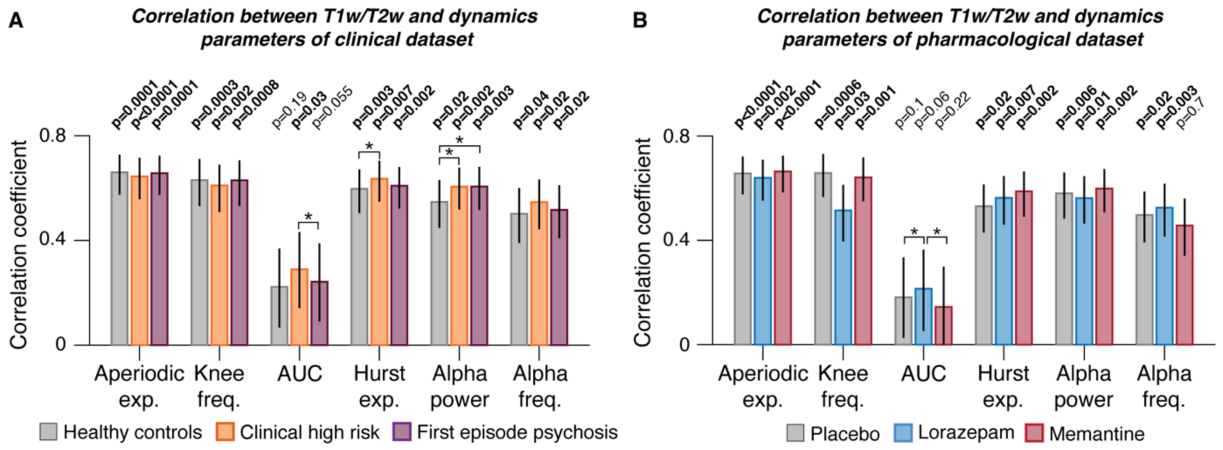

Figure S2: Anatomical hierarchy correlates with several dynamics parameters.
(A) Across-area correlations between dynamics parameters and T1w/T2w maps for clinical data (healthy controls and both clinical groups). **(B)** As panel A, but for pharmacological data (placebo and both drug conditions). Asterisks: significant difference between correlation coefficients healthy controls vs. each clinical group, or between placebo and each drug condition. Error bars are 95% bootstrap confidence intervals.

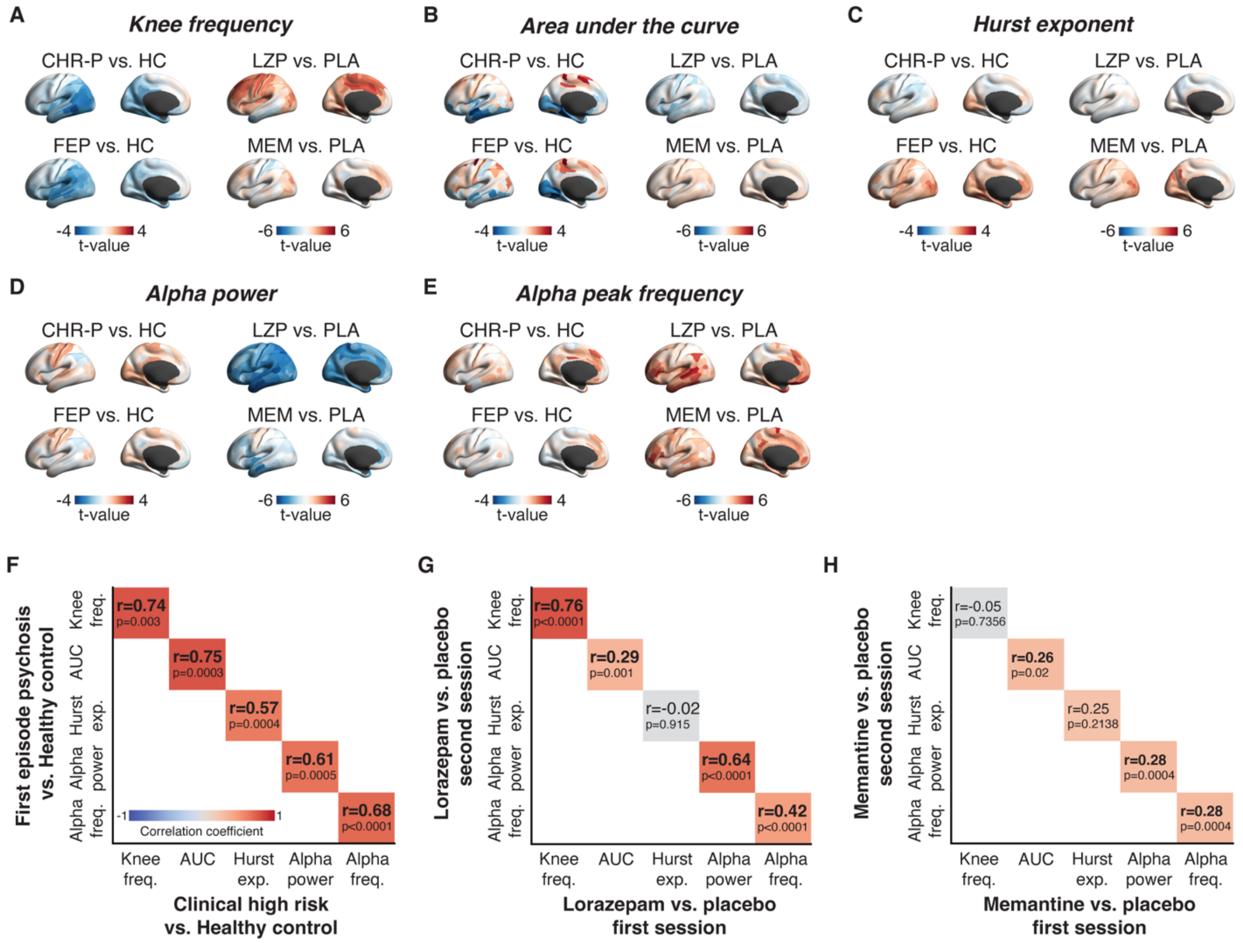

Figure S3: Heterogenous and reliable maps of psychosis signatures and drug effects in cortical dynamics.

(A-E) Maps of drug effects or psychosis signatures on five dynamics parameters (LZP: lorazepam; MEM: memantine; CHR-P: clinical high risk; FEP: first episode psychosis; HC: healthy control). Results are presented as group level non-thresholded t-values. **(A)** Knee frequency, **(B)** AUC, **(C)** Hurst exponent, **(D)** Alpha power and **(E)** Alpha peak frequency. **(F)** Spatial correlations between the psychosis signature in the clinical high risk and first episode psychosis groups. **(G, H)** Spatial correlations between drug effects in the first and second repeat of the pharmacological condition, separated by 1-5 weeks. (G) Lorazepam, (H) memantine. All correlations are Spearman's correlation coefficients and all p-values were estimated using spatial autocorrelation-preserving permutation tests.

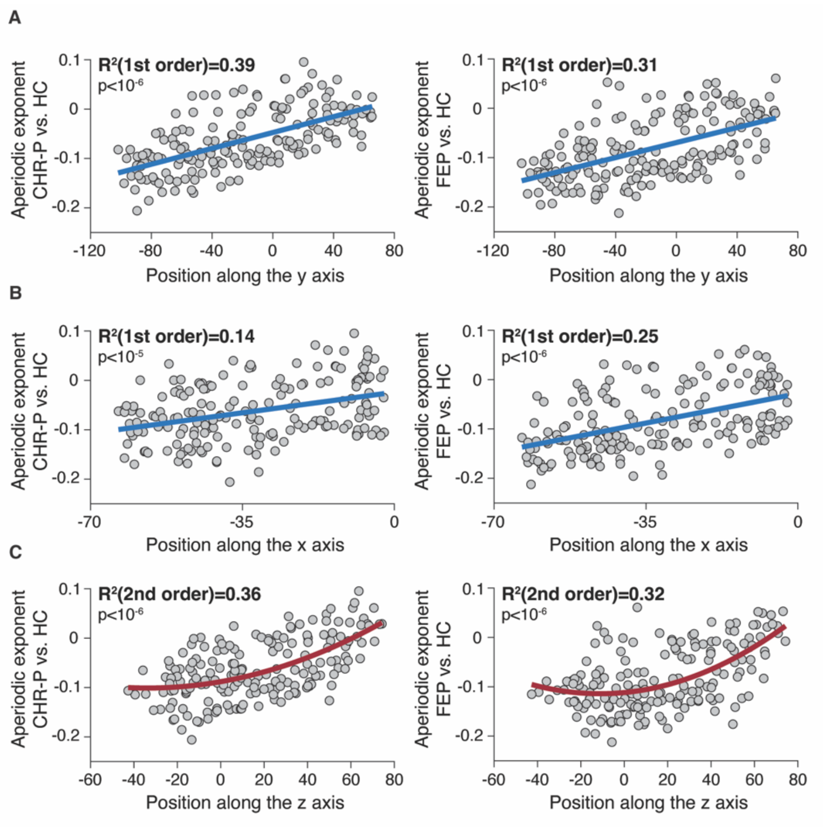

Figure S4: Variation of psychosis signature along axes of MNI space.
(A) Relation of the psychosis signature (Left: CHR vs. HC, Right: FEP vs. HC) for the aperiodic exponent with position on the y-axis in the anatomical MNI standard space. **(B-C)** same as (A) for x-axis (B), z-axis (C). Data points, cortical areas; blue line, first-order polynomial fit; red line, second-order polynomial fit; R^2 and p-values of the best-fitting model (first- or second-order polynomial fit) are shown. We performed a polynomial regression model selection (see Materials & Methods), by fitting polynomial functions with increasing order, until there was no significant improvement (R^2) of the full model, compared to the reduced model. P-values were assessed using F-test for nested models. A second order polynomial function (quadratic) indicates a non-monotonic change along the axis, and was evident for projections along the z and diagonal y+z axes (Fig. 3). A first order polynomial function (linear) best fitted along the y- and x-axes, Results were consistent across both clinical groups.

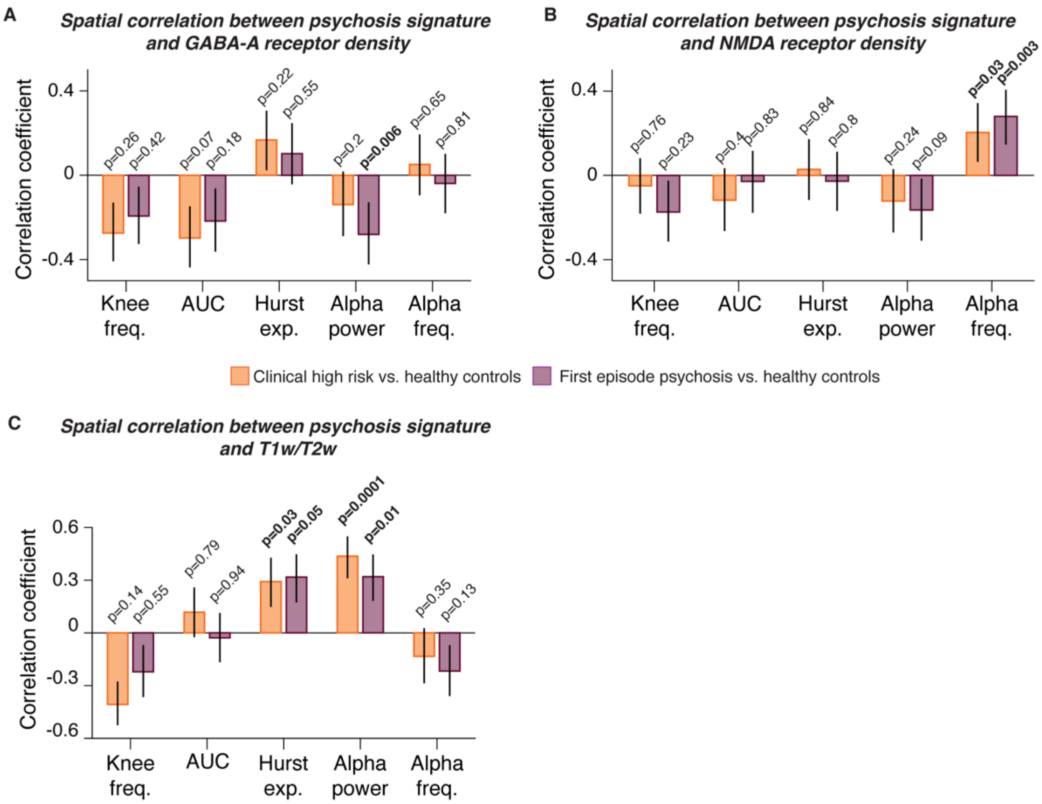

Figure S5: Similarity between maps of psychosis signatures on dynamics parameters and maps of GABA-A or NMDA receptor density levels, or T1w/T2w maps.

(A, B) Spatial correlations between the psychosis signature on dynamics parameters and GABA-A (A) or NMDA (B) receptor densities. **(C)** Spatial correlations between psychosis signature and T1w/T2w maps. Error bars are 95% bootstrap confidence interval. All correlations are Spearman's correlation coefficients; p-values were computed using spatial autocorrelation-preserving permutation tests.

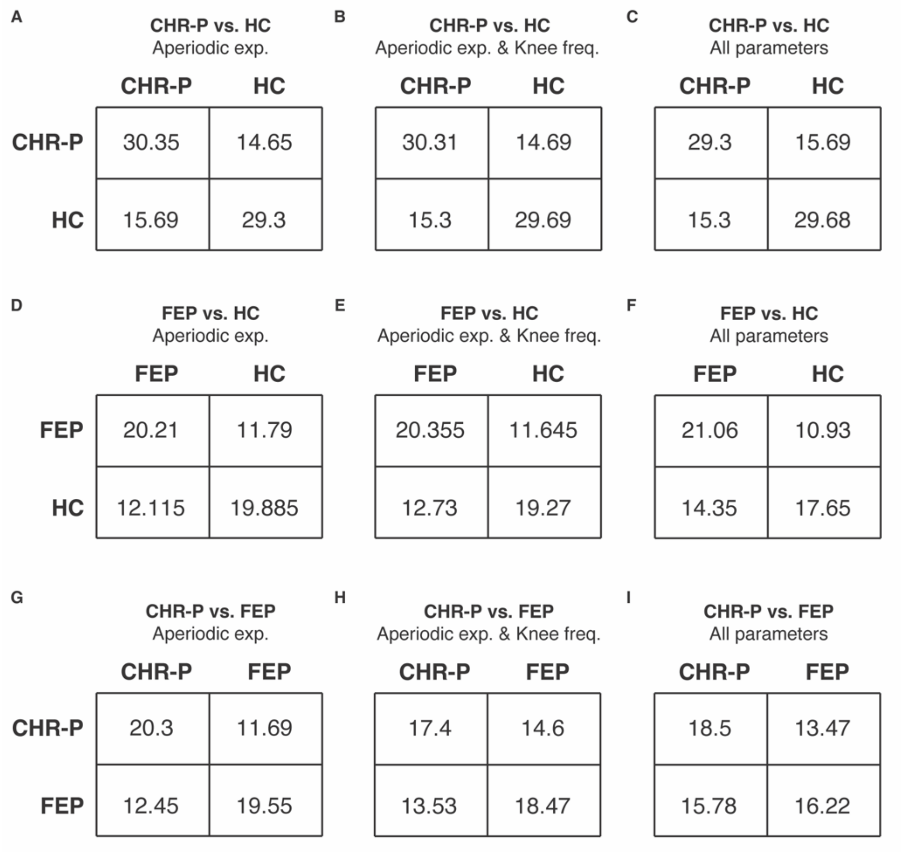

Figure S6. Confusion matrices of classification analysis.

(A-I) each panel presents the average confusion matrix of each classification problem (A-C: CHR-P vs. HC; D-F: FEP vs. HC; G-I: CHR-P vs. FEP), and each set of dynamics parameters (left column: aperiodic exponent; middle: aperiodic exponent and knee frequency; right: all dynamics parameters).

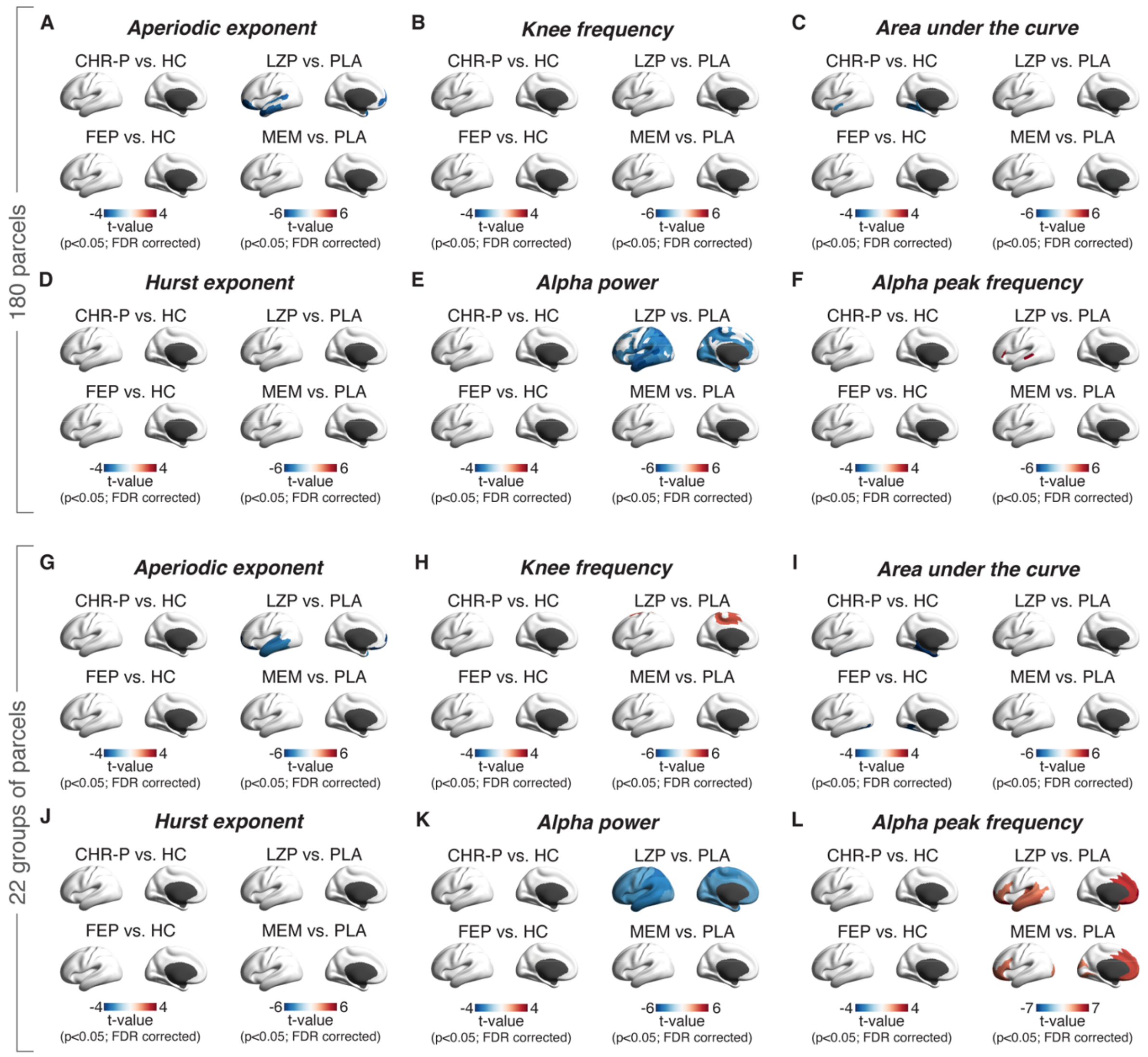

Figure S7: Weak psychosis signatures or drug effects in local cortical dynamics.
Replicas of the maps of psychosis (CHR-P vs. HC or FEP vs. HC; left) or drug effects (lorazepam vs. placebo or memantine vs. placebo; right) for different parameters from Figure S3, but after applying statistical significance threshold based on mass-univariate tests for **(A-F)** each of 180 areas or **(G-L)** for 22 groups of parcels (t-tests; $p<0.05$, FDR correction). **(A, G)** Aperiodic exponent **(B, H)** knee frequency, **(C, I)** AUC, **(D, J)** Hurst exponent, **(E, K)** alpha power, and **(F, L)** alpha peak frequency. The widespread reduction in alpha power under LZP in panels E, K is a well-documented phenomenon (*106-107*). CHR-P: clinical high risk; FEP: first episode psychosis; HC: healthy controls; LZP: lorazepam; MEM: memantine.

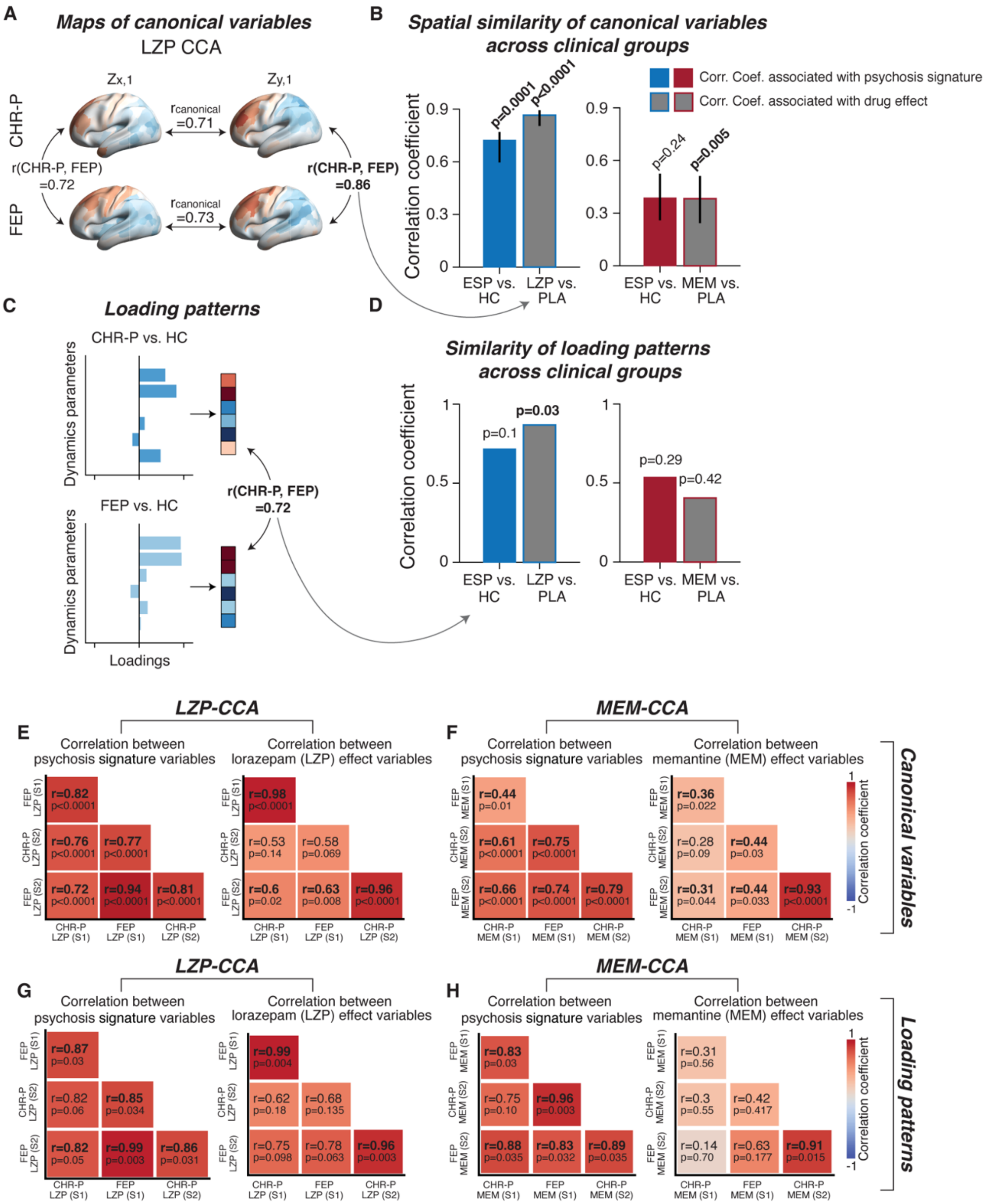

Figure S8: Reproducibility of CCA across clinical groups.

(A) Maps of the first canonical variables of the CCA between the psychosis signature (up: CHR-P vs. HC; bottom: FEP vs. HC) and lorazepam effect (LZP vs. PLA). The maps were computed separately for the CHR-P and FEP groups. Horizontal arrows: canonical correlation. Vertical arrow: spatial similarities of corresponding canonical variables across groups. **(B)** Spatial similarity of first canonical variables across the two clinical groups. ESP: Early-stage psychosis. **(C)** CCA loadings of the first canonical variable between the psychosis signature and lorazepam effect, computed separately for the CHR-P and FEP groups. Horizontal arrow: correlation between

loadings of the two clinical groups. **(D)** Similarities between loadings across the two clinical groups. Blue/red bars: similarities across canonical variables or loadings of the psychosis signature (i.e., $Z_{x,1}$), for CCA with lorazepam/memantine, respectively. Gray bars: similarities across canonical variables or loadings of the drug effect (i.e., $Z_{y,1}$). P-values were obtained using permutation tests (corrected for spatial autocorrelation). **(E-H)** Complete results from all reproducibility analyses. **(E,F)** Correlation matrices between all pairs of canonical variables, separated by drug (E: lorazepam; F: memantine), and canonical variable (left: psychosis signature; right: drug effect). All correlations are Spearman's correlation and p-values were estimated using spatial autocorrelation-preserving permutation tests. **(G,H)** same as (E,F) but for the CCA loading patterns.

REFERENCES AND NOTES

1. M. Solmi, G. Seitidis, D. Mavridis, C. U. Correll, E. Dragioti, S. Guimond, L. Tuominen, A. Dargél, A. F. Carvalho, M. Fornaro, M. Maes, F. Monaco, M. Song, J. Il Shin, S. Cortese, Incidence, prevalence, and global burden of schizophrenia – Data, with critical appraisal, from the Global Burden of Disease (GBD) 2019. *Mol. Psychiatry* **28**, 5319–5327 (2023).

2. F. J. Charlson, A. J. Ferrari, D. F. Santomauro, S. Diminic, E. Stockings, J. G. Scott, J. J. McGrath, H. A. Whiteford, Global epidemiology and burden of schizophrenia: Findings from the Global Burden of Disease Study 2016. *Schizophr. Bull.* **44**, 1195–1203 (2018).

3. T. R. Insel, Rethinking schizophrenia. *Nature* **468**, 187–193 (2010).

4. T. Insel, B. Cuthbert, M. Garvey, R. Heinssen, D. S. Pine, K. Quinn, C. Sanislow, P. Wang, Research Domain Criteria (RDoC): Toward a new classification framework for research on mental disorders. *Am. J. Psychiatry* **167**, 748–751 (2010).

5. P. Fusar-Poli, S. Borgwardt, A. Bechdolf, J. Addington, A. Riecher-Rössler, F. Schultze-Lutter, M. Keshavan, S. Wood, S. Ruhrmann, L. J. Seidman, L. Valmaggia, T. Cannon, E. Velthorst, L. De Haan, B. Cornblatt, I. Bonoldi, M. Birchwood, T. McGlashan, W. Carpenter, P. McGorry, J. Klosterkötter, P. McGuire, A. Yung, The psychosis high-risk state: A comprehensive state-of-the-art review. *Arch. Gen. Psychiatry* **70**, 107–120 (2013).

6. G. S. De Pablo, J. Radua, J. Pereira, I. Bonoldi, V. Arienti, F. Besana, L. Soardo, A. Cabras, L. Fortea, A. Catalan, J. Vaquerizo-Serrano, F. Coronelli, S. Kaur, J. Da Silva, J. I. Shin, M. Solmi, N. Brondino, P. Politi, P. McGuire, P. Fusar-Poli, Probability of transition to psychosis in individuals at clinical high risk: An updated meta-analysis. *JAMA Psychiatry* **78**, 970–978 (2021).

7. A. Carlsson, N. Waters, S. Holm-Waters, J. Tedroff, M. Nilsson, M. L. Carlsson, Interactions between monoamines, glutamate, and GABA in schizophrenia: New evidence. *Annu. Rev. Pharmacol. Toxicol.* **41**, 237–260 (2001).

8. M. Carlén, K. Meletis, J. H. Siegle, J. A. Cardin, K. Futai, D. Vierling-Claassen, C. Rühlmann, S. R. Jones, K. Deisseroth, M. Sheng, C. I. Moore, L. H. Tsai, A critical role for NMDA

receptors in parvalbumin interneurons for gamma rhythm induction and behavior. *Mol. Psychiatry* **17**, 537–548 (2012).

9. D. A. Lewis, T. Hashimoto, D. W. Volk, Cortical inhibitory neurons and schizophrenia. *Nat. Rev. Neurosci.* **6**, 312–324 (2005).

10. J. H. Yoon, R. J. Maddock, E. DongBo Cui, M. J. Minzenberg, T. A. Niendam, T. Lesh, M. Solomon, J. D. Ragland, C. Carter, Reduced in vivo visual cortex GABA in schizophrenia, a replication in a recent onset sample. *Schizophr. Res.* **215**, 217–222 (2020).

11. J. H. Yoon, R. J. Maddock, A. Rokem, M. A. Silver, M. J. Minzenberg, J. D. Ragland, C. S. Carter, GABA concentration is reduced in visual cortex in schizophrenia and correlates with orientation-specific surround suppression. *J. Neurosci.* **30**, 3777–3781 (2010).

12. J. E. Lisman, J. T. Coyle, R. W. Green, D. C. Javitt, F. M. Benes, S. Heckers, A. A. Grace, Circuit-based framework for understanding neurotransmitter and risk gene interactions in schizophrenia. *Trends Neurosci.* **31**, 234–242 (2008).

13. J. D. Murray, A. Anticevic, M. Gancsos, M. Ichinose, P. R. Corlett, J. H. Krystal, X. J. Wang, Linking microcircuit dysfunction to cognitive impairment: Effects of disinhibition associated with schizophrenia in a cortical working memory model. *Cereb. Cortex* **24**, 859–872 (2014).

14. J. H. Krystal, L. P. Karper, J. P. Seibyl, G. K. Freeman, R. Delaney, J. Douglas Bremner, G. R. Heninger, M. B. Bowers, D. S. Charney, Subanesthetic effects of the noncompetitive NMDA antagonist, ketamine, in humans: Psychotomimetic, perceptual, cognitive, and neuroendocrine responses. *Arch. Gen. Psychiatry* **51**, 199–214 (1994).

15. G. Cruz, T. Grent- ‘t-Jong, R. Krishnadas, J. M. Palva, S. Palva, P. J. Uhlhaas, Long range temporal correlations (LRTCs) in MEG-data during emerging psychosis: Relationship to symptoms, medication-status and clinical trajectory. *Neuroimage Clin.* **31**, 102722 (2021).

16. J. L. Molina, B. Voytek, M. L. Thomas, Y. B. Joshi, S. G. Bhakta, J. A. Talledo, N. R. Swerdlow, G. A. Light, Memantine effects on electroencephalographic measures of putative

excitatory/inhibitory balance in schizophrenia. *Biol. Psychiatry Cogn. Neurosci. Neuroimaging* **5**, 562–568 (2020).

17. R. J. Earl, T. C. Ford, J. A. G. Lum, P. G. Enticott, A. T. Hill, Exploring aperiodic activity in first episode schizophrenia spectrum psychosis: A resting-state EEG analysis. *Brain Res.* **1840**, 149052 (2024).

18. M. Zeev-Wolf, J. Levy, C. Jahshan, A. Peled, Y. Levkovitz, A. Grinshpoon, A. Goldstein, MEG resting-state oscillations and their relationship to clinical symptoms in schizophrenia. *Neuroimage Clin.* **20**, 753–761 (2018).

19. M. R. Goldstein, M. J. Peterson, J. L. Sanguinetti, G. Tononi, F. Ferrarelli, Topographic deficits in alpha-range resting EEG activity and steady state visual evoked responses in schizophrenia. *Schizophr. Res.* **168**, 145–152 (2015).

20. F. T. Candelaria-Cook, M. E. Schendel, C. J. Ojeda, J. R. Bustillo, J. M. Stephen, Reduced parietal alpha power and psychotic symptoms: Test-retest reliability of resting-state magnetoencephalography in schizophrenia and healthy controls. *Schizophr. Res.* **215**, 229–240 (2020).

21. Y. Hirano, P. J. Uhlhaas, Current findings and perspectives on aberrant neural oscillations in schizophrenia introduction: Schizophrenia. *Psychiatry Clin. Neurosci.* **75**, 358–368 (2021).

22. P. J. Uhlhaas, W. Singer, Abnormal neural oscillations and synchrony in schizophrenia. *Nat. Rev. Neurosci.* **11**, 100–113 (2010).

23. P. J. Uhlhaas, W. Singer, Oscillations and neuronal dynamics in schizophrenia: The search for basic symptoms and translational opportunities. *Biol. Psychiatry* **77**, 1001–1009 (2015).

24. X. J. Wang, Macroscopic gradients of synaptic excitation and inhibition in the neocortex. *Nat. Rev. Neurosci.* **21**, 169–178 (2020).

25. N. H. Lam, T. Borduqui, J. Hallak, A. Roque, A. Anticevic, J. H. Krystal, X. J. Wang, J. D. Murray, Effects of altered excitation-inhibition balance on decision making in a cortical circuit model. *J. Neurosci.* **42**, 1035–1053 (2022).

26. X. J. Wang, Decision making in recurrent neuronal circuits. *Neuron* **60**, 215–234 (2008).

27. M. N. Shadlen, W. T. Newsome, The variable discharge of cortical neurons: Implications for connectivity, computation, and information coding. *J. Neurosci.* **18**, 3870–3896 (1998).

28. J. P. Roach, A. K. Churchland, T. A. Engel, Choice selective inhibition drives stability and competition in decision circuits. *Nat. Commun.* **14**, 147 (2023).

29. T. Pfeffer, A. Ponce-Alvarez, K. Tsetsos, T. Meindertsma, C. J. Gahnström, R. Lucas Van Den Brink, G. Nolte, A. K. Engel, G. Deco, T. H. Donner, Circuit mechanisms for the chemical modulation of cortex-wide network interactions and behavioral variability. *Sci. Adv.* **7**, 5620–5636 (2021).

30. Y. Shu, A. Hasenstaub, D. A. McCormick, Turning on and off recurrent balanced cortical activity. *Nature* **423**, 283–288 (2003).

31. O. Yizhar, L. E. Fenno, M. Prigge, F. Schneider, T. J. Davidson, D. J. Ogshea, V. S. Sohal, I. Goshen, J. Finkelstein, J. T. Paz, K. Stehfest, R. Fudim, C. Ramakrishnan, J. R. Huguenard, P. Hegemann, K. Deisseroth, Neocortical excitation/inhibition balance in information processing and social dysfunction. *Nature* **477**, 171–178 (2011).

32. A. T. Kuan, G. Bondanelli, L. N. Driscoll, J. Han, M. Kim, D. G. C. Hildebrand, B. J. Graham, D. E. Wilson, L. A. Thomas, S. Panzeri, C. D. Harvey, W. C. A. Lee, Synaptic wiring motifs in posterior parietal cortex support decision-making. *Nature* **627**, 367–373 (2024).

33. J. L. R. Rubenstein, M. M. Merzenich, Model of autism: Increased ratio of excitation/inhibition in key neural systems. *Genes Brain Behav.* **2**, 255–267 (2003).

34. J. Lisman, Excitation, inhibition, local oscillations, or large-scale loops: What causes the symptoms of schizophrenia? *Curr. Opin. Neurobiol.* **22**, 537–544 (2012).

35. C. E. Robertson, S. Baron-Cohen, Sensory perception in autism. *Nat. Rev. Neurosci.* **18**, 671–684 (2017).

36. K. J. Miller, L. B. Sorensen, J. G. Ojemann, M. Den Nijs, Power-law scaling in the brain surface electric potential. *PLOS Comput. Biol.* **5**, e1000609 (2009).

37. S. S. Poil, R. Hardstone, H. D. Mansvelder, K. Linkenkaer-Hansen, Critical-state dynamics of avalanches and oscillations jointly emerge from balanced excitation/inhibition in neuronal networks. *J. Neurosci.* **32**, 9817–9823 (2012).

38. R. Gao, E. J. Peterson, B. Voytek, Inferring synaptic excitation/inhibition balance from field potentials. *Neuroimage* **158**, 70–78 (2017).

39. R. Gao, R. L. Van den Brink, T. Pfeffer, B. Voytek, Neuronal timescales are functionally dynamic and shaped by cortical microarchitecture. *eLife* **9**, e61277 (2020).

40. T. Pfeffer, A. E. Avramiea, G. Nolte, A. K. Engel, K. Linkenkaer-Hansen, T. H. Donner, Catecholamines alter the intrinsic variability of cortical population activity and perception. *PLOS Biol.* **16**, e2003453 (2018).

41. P. Martínez-Cañada, T. V. Ness, G. T. Einevoll, T. Fellin, S. Panzeri, Computation of the electroencephalogram (EEG) from network models of point neurons. *PLOS Comput. Biol.* **17**, e1008893 (2021).

42. L. Waschke, N. A. Kloosterman, J. Obleser, D. D. Garrett, Behavior needs neural variability. *Neuron* **109**, 751–766 (2021).

43. S. Baillet, Magnetoencephalography for brain electrophysiology and imaging. *Nat. Neurosci.* **20**, 327–339 (2017).

44. T. H. Donner, M. Siegel, A framework for local cortical oscillation patterns. *Trends Cogn. Sci.* **15**, 191–199 (2011).

45. S. Froudist-Walsh, T. Xu, M. Niu, L. Rapan, L. Zhao, D. S. Margulies, K. Zilles, X. J. Wang, N. Palomero-Gallagher, Gradients of neurotransmitter receptor expression in the macaque cortex. *Nat. Neurosci.* **26**, 1281–1294 (2023).

46. J. B. Burt, M. Demirtaş, W. J. Eckner, N. M. Navejar, J. L. Ji, W. J. Martin, A. Bernacchia, A. Anticevic, J. D. Murray, Hierarchy of transcriptomic specialization across human cortex captured by structural neuroimaging topography. *Nat. Neurosci.* **21**, 1251–1259 (2018).

47. N. T. Markov, J. Vezoli, P. Chameau, A. Falchier, R. Quilodran, C. Huissoud, C. Lamy, P. Misery, P. Giroud, S. Ullman, P. Barone, C. Dehay, K. Knoblauch, H. Kennedy, Anatomy of hierarchy: Feedforward and feedback pathways in macaque visual cortex. *J. Comp. Neurol.* **522**, 225–259 (2014).

48. M. F. Glasser, D. C. van Essen, Mapping human cortical areas in vivo based on myelin content as revealed by T1- and T2-weighted MRI. *J. Neurosci.* **31**, 11597–11616 (2011).

49. J. D. Murray, A. Bernacchia, D. J. Freedman, R. Romo, J. D. Wallis, X. Cai, C. Padoa-Schioppa, T. Pasternak, H. Seo, D. Lee, X. J. Wang, A hierarchy of intrinsic timescales across primate cortex. *Nat. Neurosci.* **17**, 1661–1663 (2014).

50. P. R. Murphy, N. Wilming, D. C. Hernandez-Bocanegra, G. Prat-Ortega, T. H. Donner, Adaptive circuit dynamics across human cortex during evidence accumulation in changing environments. *Nat. Neurosci.* **24**, 987–997 (2021).

51. M. Demirtaş, J. B. Burt, M. Helmer, J. L. Ji, B. D. Adkinson, M. F. Glasser, D. C. Van Essen, S. N. Sotiropoulos, A. Anticevic, J. D. Murray, Hierarchical heterogeneity across human cortex shapes large-scale neural dynamics. *Neuron* **101**, 1181–1194.e13 (2019).

52. G. Shafiei, B. D. Fulcher, B. Voytek, T. D. Satterthwaite, S. Baillet, B. Misic, Neurophysiological signatures of cortical micro-architecture. *Nat. Commun.* **14**, 6000 (2023).

53. J. Y. Hansen, G. Shafiei, R. D. Markello, K. Smart, S. M. L. Cox, M. Nørgaard, V. Beliveau, Y. Wu, J. D. Gallezot, É. Aumont, S. Servaes, S. G. Scala, J. M. DuBois, G. Wainstein, G. Bezgin, T. Funck, T. W. Schmitz, R. N. Spreng, M. Galovic, M. J. Koepp, J. S. Duncan, J. P. Coles, T. D. Fryer, F. I. Aigbirhio, C. J. McGinnity, A. Hammers, J. P. Soucy, S. Baillet, S. Guimond, J. Hietala, M. A. Bedard, M. Leyton, E. Kobayashi, P. Rosa-Neto, M. Ganz, G. M. Knudsen, N. Palomero-Gallagher, J. M. Shine, R. E. Carson, L. Tuominen, A. Dagher, B.

Misic, Mapping neurotransmitter systems to the structural and functional organization of the human neocortex. *Nat. Neurosci.* **25**, 1569–1581 (2022).

54. J. Y. Hansen, R. D. Markello, L. Tuominen, M. Nørgaard, E. Kuzmin, N. Palomero-Gallagher, A. Dagher, B. Misic, Correspondence between gene expression and neurotransmitter receptor and transporter density in the human brain. *Neuroimage* **264**, 119671 (2022).

55. M. F. Glasser, T. S. Coalson, E. C. Robinson, C. D. Hacker, J. Harwell, E. Yacoub, K. Ugurbil, J. Andersson, C. F. Beckmann, M. Jenkinson, S. M. Smith, D. C. Van Essen, A multi-modal parcellation of human cerebral cortex. *Nature* **536**, 171–178 (2016).

56. J. Da, S. Castanheira, A. I. Wiesman, J. Y. Hansen, B. Misic, S. Baillet, The neurophysiological brain-fingerprint of Parkinson's disease. *EBioMedicine* **105**, 105201 (2024).

57. A. I. Wiesman, J. da Silva Castanheira, E. A. Fon, S. Baillet, Alterations of cortical structure and neurophysiology in Parkinson's disease are aligned with neurochemical systems. *Ann. Neurol.* **95**, 802–816 (2024).

58. R. Chaudhuri, B. J. He, X. J. Wang, Random recurrent networks near criticality capture the broadband power distribution of human ECoG dynamics. *Cereb. Cortex* **28**, 3610–3622 (2018).

59. T. C. Südhof, Neuroligins and neurexins link synaptic function to cognitive disease. *Nature* **455**, 903–911 (2008).

60. P. J. Uhlhaas, R. Gajwani, J. Gross, A. I. Gumley, S. M. Lawrie, M. Schwannauer, The youth mental health risk and resilience study (YouR-Study). *BMC Psychiatry* **17**, 43 (2017).

61. A. Toso, A. P. Wermuth, A. Arazi, A. Braun, T. Grent-'t Jong, P. J. Uhlhaas, T. H. Donner, 40 Hz steady-state response in human auditory cortex is shaped by gabaergic neuronal inhibition. *J. Neurosci.* **44**, e2029232024 (2024).

62. T. Grent-‘t-Jong, R. Gajwani, J. Gross, A. I. Gumley, R. Krishnadas, S. M. Lawrie, M. Schwannauer, F. Schultze-Lutter, P. J. Uhlhaas, 40-Hz auditory steady-state responses characterize circuit dysfunctions and predict clinical outcomes in clinical high-risk for psychosis participants: A magnetoencephalography study. *Biol. Psychiatry* **90**, 419–429 (2021).

63. T. Grent-‘t-Jong, R. Gajwani, J. Gross, A. I. Gumley, R. Krishnadas, S. M. Lawrie, M. Schwannauer, F. Schultze-Lutter, P. J. Uhlhaas, Association of magnetoencephalographically measured high-frequency oscillations in visual cortex with circuit dysfunctions in local and large-scale networks during emerging psychosis. *JAMA Psychiatry* **77**, 852–862 (2020).

64. T. Grent-‘t’Jong, J. Gross, J. Goense, M. Wibral, R. Gajwani, A. I. Gumley, S. M. Lawrie, M. Schwannauer, F. Schultze-Lutter, T. N. Schröder, D. Koethe, F. M. Leweke, W. Singer, P. J. Uhlhaas, Resting-state gamma-band power alterations in schizophrenia reveal E/I-balance abnormalities across illness-stages. *eLife* **7**, e37799 (2018).

65. T. Donoghue, M. Haller, E. J. Peterson, P. Varma, P. Sebastian, R. Gao, T. Noto, A. H. Lara, J. D. Wallis, R. T. Knight, A. Shestyuk, B. Voytek, Parameterizing neural power spectra into periodic and aperiodic components. *Nat. Neurosci.* **23**, 1655–1665 (2020).

66. R. Hardstone, S. S. Poil, G. Schiavone, R. Jansen, V. V. Nikulin, H. D. Mansvelder, K. Linkenkaer-Hansen, Detrended fluctuation analysis: A scale-free view on neuronal oscillations. *Front. Physiol.* **3**, 450 (2012).

67. A. Rosenberg, J. S. Patterson, D. E. Angelaki, A computational perspective on autism. *Proc. Natl. Acad. Sci. U.S.A.* **112**, 9158–9165 (2015).

68. P. Fries, Neuronal gamma-band synchronization as a fundamental process in cortical computation. *Annu. Rev. Neurosci.* **32**, 209–224 (2009).

69. G. Buzśaki, X. J. Wang, Mechanisms of gamma oscillations. *Annu. Rev. Neurosci.* **35**, 203–225 (2012).

70. N. Wilming, P. R. Murphy, F. Meyniel, T. H. Donner, Large-scale dynamics of perceptual decision information across human cortex. *Nat. Commun.* **11**, 5109 (2020).

71. A. Goulas, J.-P. Changeux, K. Wagstyl, K. Amunts, N. Palomero-Gallagher, C. C. Hilgetag, O. Vogt, The natural axis of transmitter receptor distribution in the human cerebral cortex. *Proc. Natl. Acad. Sci. U.S.A.* **118**, e2020574118 (2021).

72. K. Zilles, K. Amunts, Receptor mapping: Architecture of the human cerebral cortex. *Curr. Opin. Neurol.* **22**, 331–339 (2009).

73. E. J. Peterson, B. Q. Rosen, A. Belger, B. Voytek, A. M. Campbell, Aperiodic neural activity is a better predictor of schizophrenia than neural oscillations. *Clin. EEG Neurosci.* **54**, 434–445 (2023).

74. D. M. Green, J. A. Swets, *Signal Detection Theory and Psychophysics* (John Wiley, 1966).

75. J. N. Rouder, P. L. Speckman, D. Sun, R. D. Morey, G. Iverson, Bayesian t tests for accepting and rejecting the null hypothesis. *Psychon. Bull. Rev.* **16**, 225–237 (2009).

76. S. R. Kay, A. Flszbeln, L. A. Qpjer, The positive and negative syndrome scale (PANSS) for schizophrenia. *Schizophr. Bull.* **13**, 271–276 (1987).

77. K. Beck, G. Hindley, F. Borgan, C. Ginestet, R. McCutcheon, S. Brugger, N. Driesen, M. Ranganathan, D. C. D'Souza, M. Taylor, J. H. Krystal, O. D. Howes, Association of ketamine with psychiatric symptoms and implications for its therapeutic use and for understanding schizophrenia: A systematic review and meta-analysis. *JAMA Netw. Open* **3**, e204693 (2020).

78. J. Frohlich, J. D. Van Horn, Reviewing the ketamine model for schizophrenia. *J. Psychopharmacol.* **28**, 287–302 (2014).

79. P. R. Corlett, G. D. Honey, J. H. Krystal, P. C. Fletcher, Glutamatergic model psychoses: Prediction error, learning, and inference. *Neuropsychopharmacology* **36**, 294–315 (2011).

80. X. J. Wang, J. H. Krystal, Computational psychiatry. *Neuron* **84**, 638–654 (2014).

81. A. Anticevic, P. R. Corlett, M. W. Cole, A. Savic, M. Gancsos, Y. Tang, G. Repovs, J. D. Murray, N. R. Driesen, P. T. Morgan, K. Xu, F. Wang, J. H. Krystal, N-methyl-D-aspartate receptor antagonist effects on prefrontal cortical connectivity better model early than chronic schizophrenia. *Biol. Psychiatry* **77**, 569–580 (2015).

82. J. D. Haynes, G. Rees, Decoding mental states from brain activity in humans. *Nat. Rev. Neurosci.* **7**, 523–534 (2006).

83. N. Kriegeskorte, P. Bandettini, Analyzing for information, not activation, to exploit high-resolution fMRI. *Neuroimage* **38**, 649–662 (2007).

84. J. D. Haynes, G. Rees, Predicting the orientation of invisible stimuli from activity in human primary visual cortex. *Nat. Neurosci.* **8**, 686–691 (2005).

85. Y. Kamitani, F. Tong, Decoding the visual and subjective contents of the human brain. *Nat. Neurosci.* **8**, 679–685 (2005).

86. H. Nili, C. Wingfield, A. Walther, L. Su, W. Marslen-Wilson, N. Kriegeskorte, A toolbox for representational similarity analysis. *PLOS Comput. Biol.* **10**, e1003553 (2014).

87. I. S. Ramsay, P. Lynn, B. Schermitzler, S. Sponheim, Individual alpha peak frequency is slower in schizophrenia and related to deficits in visual perception and cognition. *Sci. Rep.* **11**, 17852 (2021).

88. J. Veit, R. Hakim, M. P. Jadi, T. J. Sejnowski, H. Adesnik, Cortical gamma band synchronization through somatostatin interneurons. *Nat. Neurosci.* **20**, 951–959 (2017).

89. M. Steriade, Grouping of brain rhythms in corticothalamic systems. *Neuroscience* **137**, 1087–1106 (2006).

90. R. V. Raut, A. Z. Snyder, M. E. Raichle, Hierarchical dynamics as a macroscopic organizing principle of the human brain. *Proc. Natl. Acad. Sci. U.S.A.* **117**, 20890–20897 (2020).

91. K. Mahjoory, J. M. Schoffelen, A. Keitel, J. Gross, The frequency gradient of human resting-state brain oscillations follows cortical hierarchies. *eLife* **9**, e53715 (2020).

92. B. C. Bernhardt, J. Smallwood, S. Keilholz, D. S. Margulies, Gradients in brain organization. *Neuroimage* **251**, 118987 (2022).

93. G. Shafiei, R. D. Markello, R. V. De Wael, B. C. Bernhardt, B. D. Fulcher, B. Misic, Topographic gradients of intrinsic dynamics across neocortex. *eLife* **9**, e62116 (2020).

94. R. Christopher Decharms, A. Zador, Neural representation and the cortical code. *Annu. Rev. Neurosci.* **23**, 613–647 (2020).

95. A. R. Yung, P. Yuen, P. D. Mcgorry, L. J. Phillips, D. Kelly, M. Dell'olio, S. M. Francey, E. M. Cosgrave, E. Killackey, C. Stanford, K. Godfrey, J. Buckby, Mapping the onset of psychosis: The comprehensive assessment of at-risk mental states. *Aust. N. Z. J. Psychiatry* **39**, 964–971 (2005).

96. F. Schultze-Lutter, J. Addington, S. Ruhrmann, J. Klosterkötter, *Schizophrenia Proneness Instrument, Adult Version (SPI-A)* (Giovanni Fioriti Editore, RomE, 2007).

97. J. W. Johnson, S. E. Kotermanski, Mechanism of action of memantine. *Curr. Opin. Pharmacol.* **6**, 61–67 (2006).

98. L. Waschke, T. Donoghue, L. Fiedler, S. Smith, D. D. Garrett, B. Voytek, J. Obleser, Modality-specific tracking of attention and sensory statistics in the human electrophysiological spectral exponent. *eLife* **10**, e70068 (2021).

99. S. V. Salvatore, P. M. Lambert, A. Benz, N. R. Rensing, M. Wong, C. F. Zorumski, S. Mennerick, Periodic and aperiodic changes to cortical EEG in response to pharmacological manipulation. *J. Neurophysiol.* **131**, 529–540 (2024).

100. R. Oostenveld, P. Fries, E. Maris, J. M. Schoffelen, FieldTrip: Open source software for advanced analysis of MEG, EEG, and invasive electrophysiological data. *Comput. Intell. Neurosci.* **2011**, 156869 (2011).

101. A. Gramfort, M. Luessi, E. Larson, D. A. Engemann, D. Strohmeier, C. Brodbeck, R. Goj, M. Jas, T. Brooks, L. Parkkonen, M. Hämäläinen, MEG and EEG data analysis with MNE-Python. *Front. Neurosci.* **7**, 267 (2013).

102. M. Gerster, G. Waterstraat, V. Litvak, K. Lehnertz, A. Schnitzler, E. Florin, G. Curio, V. Nikulin, Separating neural oscillations from aperiodic 1/f activity: Challenges and recommendations. *Neuroinformatics* **20**, 991–1012 (2022).

103. A. F. Alexander-Bloch, H. Shou, S. Liu, T. D. Satterthwaite, D. C. Glahn, R. T. Shinohara, S. N. Vandekar, A. Raznahan, On testing for spatial correspondence between maps of human brain structure and function. *Neuroimage* **178**, 540–551 (2018).

104. R. D. Markello, B. Misic, Comparing spatial null models for brain maps. *Neuroimage* **236**, 118052 (2021).

105. J. Y. Hansen, R. D. Markello, J. W. Vogel, J. Seidlitz, D. Bzdok, B. Misic, Mapping gene transcription and neurocognition across human neocortex. *Nat. Hum. Behav.* **5**, 1240–1250 (2021).

106. J. Ahveninen, F. H. Lin, R. Kivisaari, T. Autti, M. Hämäläinen, S. Stufflebeam, J. W. Belliveau, S. Kähkönen, MRI-constrained spectral imaging of benzodiazepine modulation of spontaneous neuromagnetic activity in human cortex. *Neuroimage* **35**, 577–582 (2007).

107. M. Schreckenberger, C. Lange-Asschenfeld, M. Lochmann, K. Mann, T. Siessmeier, H. G. Buchholz, P. Bartenstein, G. Gründer, The thalamus as the generator and modulator of EEG alpha rhythm: A combined PET/EEG study with lorazepam challenge in humans. *Neuroimage* **22**, 637–644 (2004).
